# Global burden of disease 2021: particulate matter pollution–attributable burden of upper respiratory infections, otitis media, and lower respiratory infections

**DOI:** 10.3389/fpubh.2026.1666976

**Published:** 2026-03-12

**Authors:** Chaochao Wei, Yue Tang, Yang Wang

**Affiliations:** 1 Department of Pulmonary and Critical Care Medicine, Hainan General Hospital, Haikou, China; 2 Department of Pulmonary and Critical Care Medicine, Hainan Affiliated Hospital of Hainan Medical University, Haikou, China; 3 Department of Oncology, Xiangya Hospital Central South University, Changsha, China; 4 NHC Key Laboratory of Tropical Disease Control, Hainan Medical University, Haikou, China; 5 Department of Design, School of Architecture and Art, Central South University, Changsha, Hunan, China; 6 Department of Geriatrics, The Second Xiangya Hospital of Central South University, Changsha, Hunan, China

**Keywords:** burden, DALYs, particulate matter pollution, lower respiratory infections, upper respiratory infections, otitis media

## Abstract

The global burden of lower respiratory infections (LRIs), upper respiratory infections (URIs), and otitis media attributable to air pollution has exhibited notable temporal patterns. Our study analyzed the patterns in disability-adjusted life years (DALYs) and age-standardized DALY rates (ASDRs) attributable to particulate matter pollution (PMP) for LRIs, and for URIs and otitis media (infants <1 year), using Global Burden of Disease (GBD) 1990–2021 estimates. We applied frontier analysis to estimate improvement potential by development status, assessed cross-country inequality, and used decomposition analysis to evaluate the contributions of population growth, aging, and epidemiological changes. Furthermore, an autoregressive integrated moving average (ARIMA) model was employed to forecast trends through 2031. Our findings revealed that the PMP -attributable burden of LRIs, infant URIs and infant otitis media, decreased globally from 1990 to 2021, with notable reductions in East Asia (LRIs, URIs), and Central Europe (otitis media). Despite progress, burdens remains highest in low-socio-demographic index (SDI) regions, indicating substantial potential for further reduction. Decomposition attributed most global declines to epidemiological change, whereas population growth increased burden in low-SDI regions. Projections suggest continued declines for PMP -attributable burden of LRIs and infant URIs but a slight rise for infant otitis media by 2031. These findings highlight the need for further targeted preventive interventions, especially in high-burden regions.

## Introduction

The upper respiratory tract serves as the primary defense against harmful pathogens in the respiratory system. Upper respiratory infections (URIs) are a leading cause of acute disease globally (1), with otitis media—a frequent complication among children under 5 years old—commonly prompting healthcare visits (2). URIs and otitis media have diverse etiologies, with a range of pathogens, including viruses (e.g., rhinoviruses, coronaviruses, and influenza) and bacteria (e.g., *Streptococcus pyogenes*), often in combination (3). In addition, lower respiratory infections (LRIs), defined as clinician-diagnosed pneumonia or bronchiolitis, remain a leading cause of global disability-adjusted life years (DALYs) (4). *Streptococcus pneumoniae* has emerged as the leading pathogen responsible for the highest burden of LRIs, followed by *Staphylococcus aureus* and *Klebsiella pneumoniae* (4). In particular, air pollutants may compromise host defenses against microbial pathogens (5), making it a modifiable risk factor for respiratory infections.

Air pollution consists of a mixture of gases (such as ambient ozone and nitrogen dioxide), liquids, and particulate matter from both household and ambient sources (6). As the second-leading environmental risk after tobacco, air pollution contributes to LRIs and URIs (7, 8). Particulate matter (PM), particularly fine particles with an aerodynamic diameter ≤2.5 μm (PM2.5), is primarily derived from residential fuel combustion, industrial emissions, and power generation (9). In 2019, household air pollution was responsible for an estimated 86 million years of healthy life lost, with the greatest burden falling on women and children due to their prolonged exposure to harmful smoke (10). In 2021, particulate matter was the leading air-pollution contributor, accounting for 8.0% of total DALYs (11). Specifically, PM remained an important risk factor for URIs and otitis media in 2021 (3), and was linked to higher pediatric URI admissions (12). Although PM2.5 exposure increases LRI risk via epithelial barrier disruption and inflammation, the PM2.5-related LRIs burden declined globally 1990 to 2019 (13). Even so, these observations emphasize the significant contribution of particulate matter pollution (PMP) to the global burden of LRIs, URIs, and otitis media.

The World Bank commissioned the Global Burden of Disease (GBD) study to assess disease burden and risk factors systematically (14, 15). While a previous study utilized GBD 2019 data to assess the global burden of LRIs related to PM2.5 exposure (13), the recent update of the GBD database including data for 2020 and 2021, enhances its representativeness and timeliness. Prior GBD 2021 analyses described the global burden of URIs and otitis media were predominantly all-age; even when neonatal and pediatric peaks were noted (3), infant-specific PM-attributable burden remains limited. We address this gap and further apply novel data and alternative statistical methods to analyze temporal trends in the burden attributable to PMP worldwide. These findings are expected to mitigate particulate matter pollution and reducing its associated burden of LRIs, URIs, and otitis media.

## Materials and methods

### Data sources

Our study aimed to comprehensively explore the DALYs of LRIs, URIs, and otitis media attributable to PMP from 1990 to 2021 via GBD 2021 data. The data on URIs and otitis media attributable to PMP from 1990 to 2021 are available only for infants (<1 year) in the GBD 2021 database. DALYs were evaluated by combining years of life lost due to premature death (YLLs) and years lived with disability (YLDs) by location, age, sex, year, and cause. Furthermore, the socio-demographic index (SDI) is employed to assess the sociodemographic development of a country or territory via three components: the fertility rate in females under 25 years, the schooling attainment of individuals over 15 years, and lag-distributed income per capita. SDI values are categorized into five quintiles: low, low-middle, middle, high-middle, and high (16).

### Definitions

The GBD study defines LRIs as pneumonia or bronchiolitis. The diagnostic codes for LRIs include 079.82, 466–469, 470.0, 480–481.9, 482.0–482.89, 483.0–483.9, 484.1–484.2, 484.6–484.7, 487–490.9, 510–511.9, and 513.0–513.9 in the ICD-9 diagnostics criteria, and A48.1, A70, B96.0–96.1, B97.21, B97.4–B97.6, J09–J11.89, J12–J13.9, J14–J14.0, J15–J15.8, J20–J21.9, J85.1, J91.0, P23.0–P23.4, and U04–U04.9 in the ICD-10 diagnostic criteria, excluding tuberculosis or COVID-19.^1^ The GBD study defines URIs as conditions encompassing cough, acute nasopharyngitis, sinusitis, pharyngitis, tonsillitis, laryngitis/tracheitis, epiglottitis, rhinitis, rhinosinusitis, rhinopharyngitis, supraglottitis, and the common cold. For the classification of URIs, the relevant ICD-10 codes are J00-J02, J02.8-J03, J03.8-J06.9, and J36-J36.0, while ICD-9 codes include 460–465.9, 476.9, and 475–475.9.^2^ Otitis media refers to an infection of the middle ear, including acute otitis media, and chronic otitis media. The relevant ICD-10 codes are H65-H75.83, and ICD-9 codes are 381–384.9.^3^ PMP encompasses both ambient particulate matter pollution and household air pollution. Ambient particulate matter pollution, is calculated from the population-weighted annual mean concentration of airborne PM₂.₅ (μg/m^3^). Household air pollution is defined based on the proportion of individuals utilizing solid fuel for cooking and the corresponding PM₂.₅ exposure levels.^4^

### Frontier analysis

Frontier analysis delineates the nonlinear relationship between the SDI and disease burden across countries or territories by quantifying the disparity between the current burden and its theoretical minimum. LOESS (Locally Estimated Regression) is a nonparametric smoothing technique that fits local polynomial regressions to subsets of data in order to model variable relationships (17, 18). We applied this method to explore the nonlinear association between SDI and age-standardized DALY rate (ASDR) (19). To ensure robustness, 500 bootstrap samples were performed to estimate the average age-standardized rate (ASR) for each SDI value. The absolute distance between each country’s or territory’s 2021 ASDR and the frontier line was calculated to assess the potential for improvement (20).

### Decomposition analysis

We utilized the decomposition method developed by Das Gupta to systematically examine the contributions of population growth, aging, and epidemiological changes (epidemiologic changes is age-standardized DALY rates) to the PMP-attributable burden. This approach utilizes mathematical techniques to disaggregate overall changes into distinct components, enabling the quantification of each factor’s specific contribution. We report shares (%) as each component’s ΔDALYs divided by the net change in DALYs. Consequently, values may exceed 100% when one component overcompensates the others; negative values indicate opposition to the net change.

### Cross-country inequality analysis

In this study, we employed absolute and relative inequality—the slope index of inequality (SII) and the concentration index—to assess the distributional inequality in the PMP-attributable burden of LRIs, and of URIs and otitis media among infants across countries or territories (21). The SII, representing absolute inequality, was computed by regressing national DALY rates for the total population on a relative position scale associated with sociodemographic development. The concentration index (CI), reflecting relative inequality, was derived through numerical integration of the area under the Lorenz curve, which was generated using the cumulative DALY and the corresponding cumulative population distribution ranked by the SDI (22). A negative value of the SII or concentration index indicates that a higher SDI is associated with a lower ASDR, and vice versa.

### Predictive analysis

The autoregressive integrated moving average (ARIMA) model, a time series analysis technique, was employed to forecast the future burden in the next decades. This model integrates autoregression (AR), differencing (I), and moving average (MA) components to forecast from historical data (23), which is less prone to overfitting than higher-complexity alternatives. In the ARIMA (p, d, q) model, “p” represents the number of autoregressive terms, “d” indicates the order of differencing, and “q” defines moving average terms. The optimal ARIMA model parameters (p, d, q) were selected using the auto.arima() function from the forecast package in R. Model fit was evaluated by assessing the consistency between the observed and predicted values. Residual diagnostics in Supplementary Figures S1–S3, including Q-Q plots, as well as autocorrelation function (ACF) and partial autocorrelation function (PACF) plots, were used to assess whether the residuals followed a normal distribution. The Ljung-Box test was conducted to evaluate the robustness of the residuals, confirming that the model was with randomness (white noise). The ACF and the PACF were used to guide identification of appropriate parameters (p and q). The parameter d involves the difference calculation between adjacent observations to achieve stationarity. We selected the best ARIMA (p, d, q) models with the lowest Akaike information criterion (AIC)/Bayesian information criterion (BIC) (Supplementary Table S4) and satisfactory residual diagnostics to predict the disease burden (24).

The Bayesian age-period-cohort (BAPC) model, incorporating age, period, and cohort effects, is used to project trends in LRIs attributable to PMP from 2022 to 2046. It applies generalized linear models to explore associations, and employs a Bayesian framework integrating prior sample data and information for parameter estimation (25).

### Statistical analysis

The age-standardized rate (ASR) was weighted by the corresponding proportions of the GBD standard population, and the weighted rates were then summed to obtain the ASR per 100,000 population (26). The estimates and their 95% uncertainty intervals (UIs) for DALYs were obtained from GBD 2021. Estimated annual percentage change (EAPC) is a more precise parameter for evaluating trends in age-standardized DALY rate (ASDR) over time. The EAPC was estimated via the formula 100 * (*e*^β^ − 1) and reported with a 95% confidence interval (95% CI), where β denotes the slope derived from the log-linear regression model. The ASDR was considered to have increased if both the EAPC estimates and the lower bound of the 95% CI were greater than 0. Conversely, a decrease in ASDR was considered if both the EAPC estimates and the upper bound of the 95% CI were less than 0. Otherwise, the ASDR was considered stable over time (27). R software (version 4.4.0) was used for database organization, and analysis.

## Results

### Global trends in PMP-attributable LRIs, infant URIs, and infant otitis media

Between 1990 and 2021, PMP-attributable ASDRs of LRIs, infant URIs, and infant otitis media decreased globally. For LRIs, the number of PMP-attributable DALYs declined from 87,315,640 (95% UI: 20,780,746–139,682,971) to 29,098,331 (95% UI: 6,988,265–48,127,683), and the ASDR decreased from 1462.2 (95% UI: 340.59–2338.31) per 100,000 to 420.09 (95% UI: 106.35–693.12) per 100,000 globally (Supplementary Table S1). For infant URIs, the number of PMP-attributable DALYs saw a decrease from 23781.75 (95% UI: 6477.74–48147.78) to 11708.46 (95% UI: 2800.5–28081.79), and ASDR decreased from 0.37 (95% UI: 0.1–0.75) per 100,000 to 0.19 (95% UI: 0.05–0.45) per 100,000 globally (Supplementary Table S2). For infant otitis media, the number of PMP-attributable DALYs declined from 1363.14 (95% UI: 512.18–2946.84) to 327.40 (95% UI: 156.27–626.36), and ASDR decreased from 0.02 (95% UI: 0.01–0.05) per 100,000 to 0.01 (95% UI: 0–0.01) per 100,000 globally (Supplementary Table S3). The EAPCs for the ASDRs from 1990 to 2021 were −3.76 (95% CI: −3.96 to 3.55) for PMP-attributable LRIs, −2.32 (95% CI: −2.39 to 2.25) for PMP-attributable URIs in infants, and −4.54 (95% CI: −4.8 to 4.28) for PMP-attributable otitis media in infants (Supplementary Tables S1–S3). Overall, global trends in the PMP-attributable burden of LRIs, infant URIs, and infant otitis media are similar, showing declines in both numbers of DALYs and ASDRs.

### Age, sex, and spatiotemporal patterns

**Figure 1:**
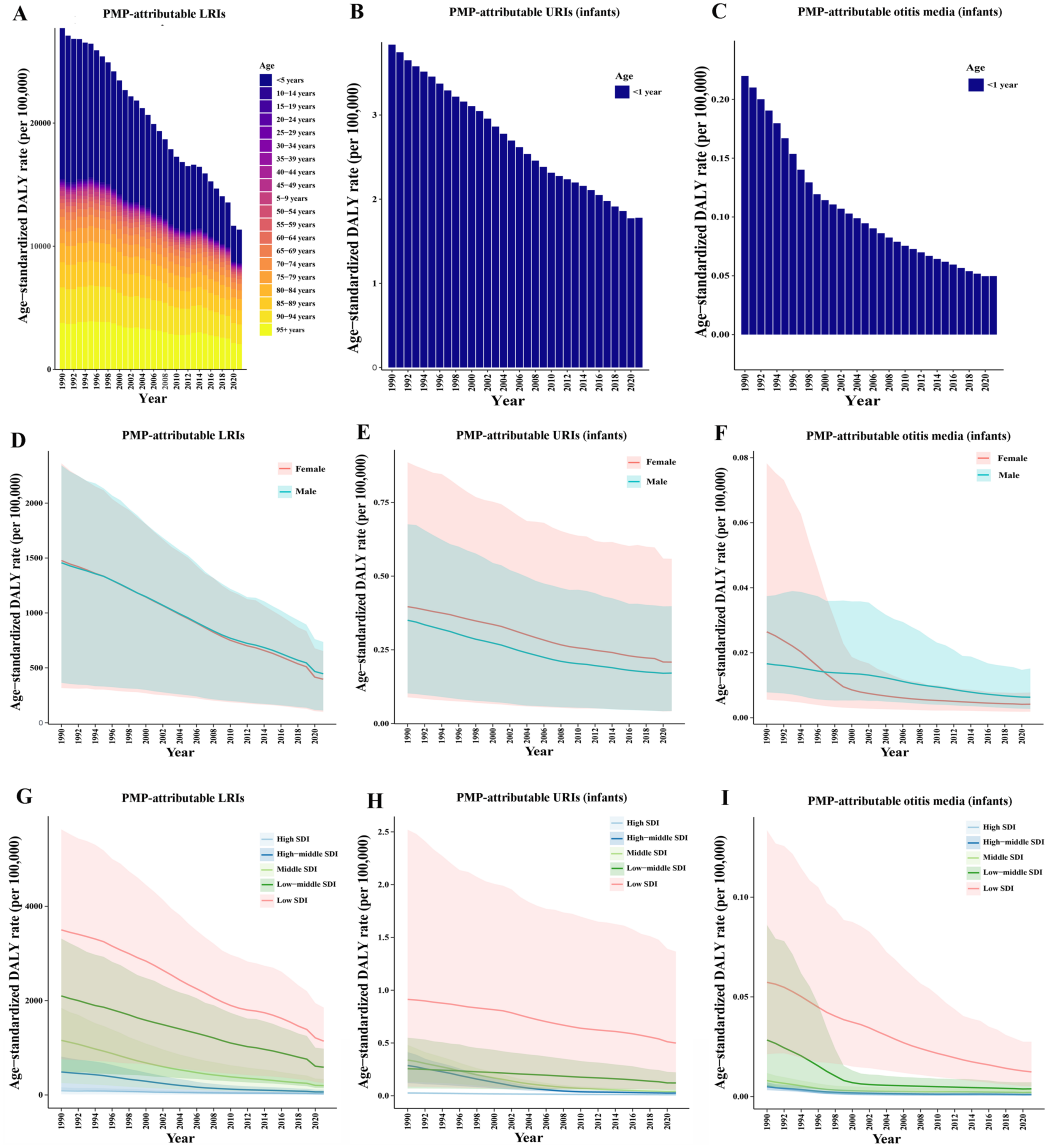
Trends in the PMP-attributable ASDRs of LRIs **(A)**, and of URIs **(B)** and otitis media **(C)** among children under 1 year by age group from 1990 to 2021. Trends in the PMP-attributable ASDRs of LRIs **(D)**, and of URIs **(E)** and otitis media **(F)** among children under 1 year in females and males from 1990 to 2021. Trends in the PMP-attributable ASDRs of LRIs **(G)**, and of URIs **(H)** and otitis media **(I)** among children under 1 year across different SDI quintiles from 1990 to 2021.

The age patterns of global DALYs for LRIs attributable to PMP are displayed in Table 1, and Figure 1A. The age group under 5 years and above 95 years presented the highest ASDR of PMP-attributable LRIs. From 1990 to 2021, all age groups displayed reductions in the ASDRs of LRIs attributable to PMP, while infants showed declining ASDRs for URIs and otitis media attributable to PMP (Figures 1A–C). From 1990 to 2021, both males and females presented a decreasing trend in the PMP-attributable ASDRs of LRIs, infant URIs, and infant otitis media (Figures 1D–F).

**Table 1:** The contributions of aging, population growth, and epidemiological trends on changes in PMP-attributable DALYs of LRIs, infant URIs, and infant otitis media.

Cause	Location	Measure	Overall difference	Change due to population-level determinants		
				(% contribute to the total changes)		
				Aging	Population	Epidemiological change
LRIs attributable to PMP	Global	DALYs	−582.17 (−100%)	−131.19 (−22.53%)	243.96 (41.9%)	−694.94 (−119.37%)
	High SDI		−3.38 (−100%)	2.55 (75.48%)	1.56 (46.32%)	−7.49 (−221.8%)
	High-middle SDI		−35.18 (−100%)	−3.81 (−10.84%)	5.44 (15.45%)	−36.8 (−104.61%)
	Middle SDI		−169.41 (−100%)	−31.22 (−18.43%)	46.37 (27.37%)	−184.57 (−108.94%)
	Low-middle SDI		−231.09 (−100%)	−65.77 (−28.46%)	119.91 (51.89%)	−285.23 (−123.43%)
	Low SDI		−142.85 (−100%)	−40.32 (−28.23%)	201.36 (140.96%)	−303.88 (−212.73%)
URIs attributable to PMP (<1 year)	Global	DALYs	−0.12 (−100%)	0 (0)	0.01 (8.89%)	−0.13 (−108.89%)
	High SDI		0 (−100%)	0 (0)	0 (−13.11%)	0 (−86.89%)
	High-middle SDI		−0.02 (−100%)	0 (0)	0 (−14.09%)	−0.02 (−85.91%)
	Middle SDI		−0.06 (−100%)	0 (0)	0 (−7.28%)	−0.06 (−92.72%)
	Low-middle SDI		−0.02 (−100%)	0 (0)	0 (14.38%)	−0.03 (−114.38%)
	Low SDI		−0.01 (−100%)	0 (0)	0.06 (594.78%)	−0.07 (−694.78%)
Otitis media attributable to PMP (<1 year)	Global	DALYs	−0.01 (−100%)	0 (0)	0 (4.98%)	−0.01 (−104.98%)
	High SDI		0 (−100%)	0 (0)	0 (−8.27%)	0 (−91.73%)
	High-middle SDI		0 (−100%)	0 (0)	0 (−17.08%)	0 (−82.92%)
	Middle SDI		0 (−100%)	0 (0)	0 (−9.14%)	0 (−90.86%)
	Low-middle SDI		0 (−100%)	0 (0)	0 (6.8%)	0 (−106.8%)
	Low SDI		−0.01 (−100%)	0 (0)	0 (76.11%)	−0.01 (−176.11%)

From 1990 to 2021, the PMP-attributable ASDRs for LRIs, infant URIs, and infant otitis media decreased across different SDI quintiles, and the low-SDI region presented the highest ASDR over time (Figures 1G–I). Regionally, Western Sub-Saharan Africa, Eastern Sub-Saharan Africa, and Central Sub-Saharan Africa had higher ASDRs in 2021 for LRIs, infant URIs, and infant otitis media, all attributable to PMP (Supplementary Tables S1–S3). The largest decrease in the PMP-attributable burden of LRIs, and of infant URIs occurred in East Asia (EAPC of LRIs = −9.13 (95% CI: −9.45 to 8.81), EAPC of URIs = −10.99 (−11.53 to 10.44), Supplementary Tables S1, S2). The largest decrease in the PMP-attributable burden of infant otitis media occurred in Central Europe [EAPC = −11.27 (−13.62 to 8.85), Supplementary Table S3].

**Figure 2:**
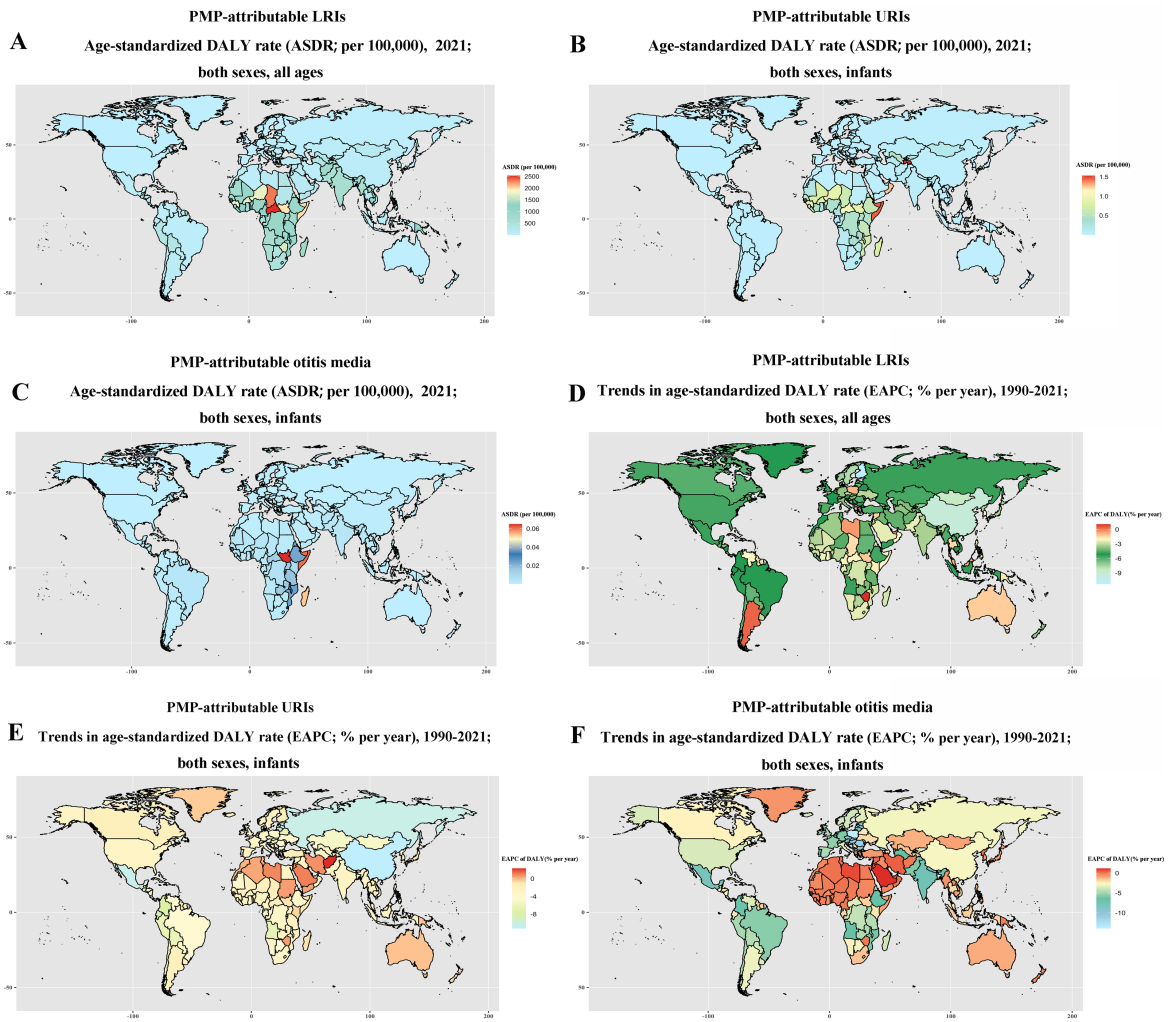
PMP-attributable burden of LRIs, infant URIs, and infant otitis across 204 countries or territories. PMP-attributable ASDRs of LRIs **(A)**, infant URIs **(B)**, and infant otitis media **(C)** in 2021; PMP-attributable EAPCs in the DALYs of LRIs **(D)**, infant URIs **(E)**, and infant otitis media **(F)** from 1990 to 2021. ASDR was calculated using the corresponding GBD standard population. Temporal trends were summarized by EAPC.

With respect to countries or territories, Central African Republic, Chad, and South Sudan showed the highest ASDRs for LRIs attributable to PMP in 2021 (Figure 2A). Tajikistan, Somalia, Oman, and Central African Republic showed the highest ASDRs for infant URIs attributable to PMP in 2021 (Figure 2B). South Sudan, Somalia, and Madagascar showed the highest ASDRs for infant otitis media attributable to PMP in 2021 (Figure 2C). From 1990 to 2021, the largest decrease in the burden of LRIs attributable to PMP occurred in Finland, China, and Ireland, whereas the largest increase occurred in Argentina, Kuwait, and Lesotho (Figure 2D). From 1990 to 2021, the largest decrease in the DALYs of infant URIs attributable to PMP occurred in China, Latvia, and Estonia, while the largest increase occurred in Afghanistan, Qatar, and Kuwait (Figure 2E). From 1990 to 2021, the largest decrease in the DALYs of infant otitis media attributable to PMP occurred in Poland, Albania, and Bosnia and Herzegovina, while the largest increase occurred in Saudi Arabia, and Kuwait (Figure 2F).

### Frontier analysis of disease burden globally

**Figure 3:**
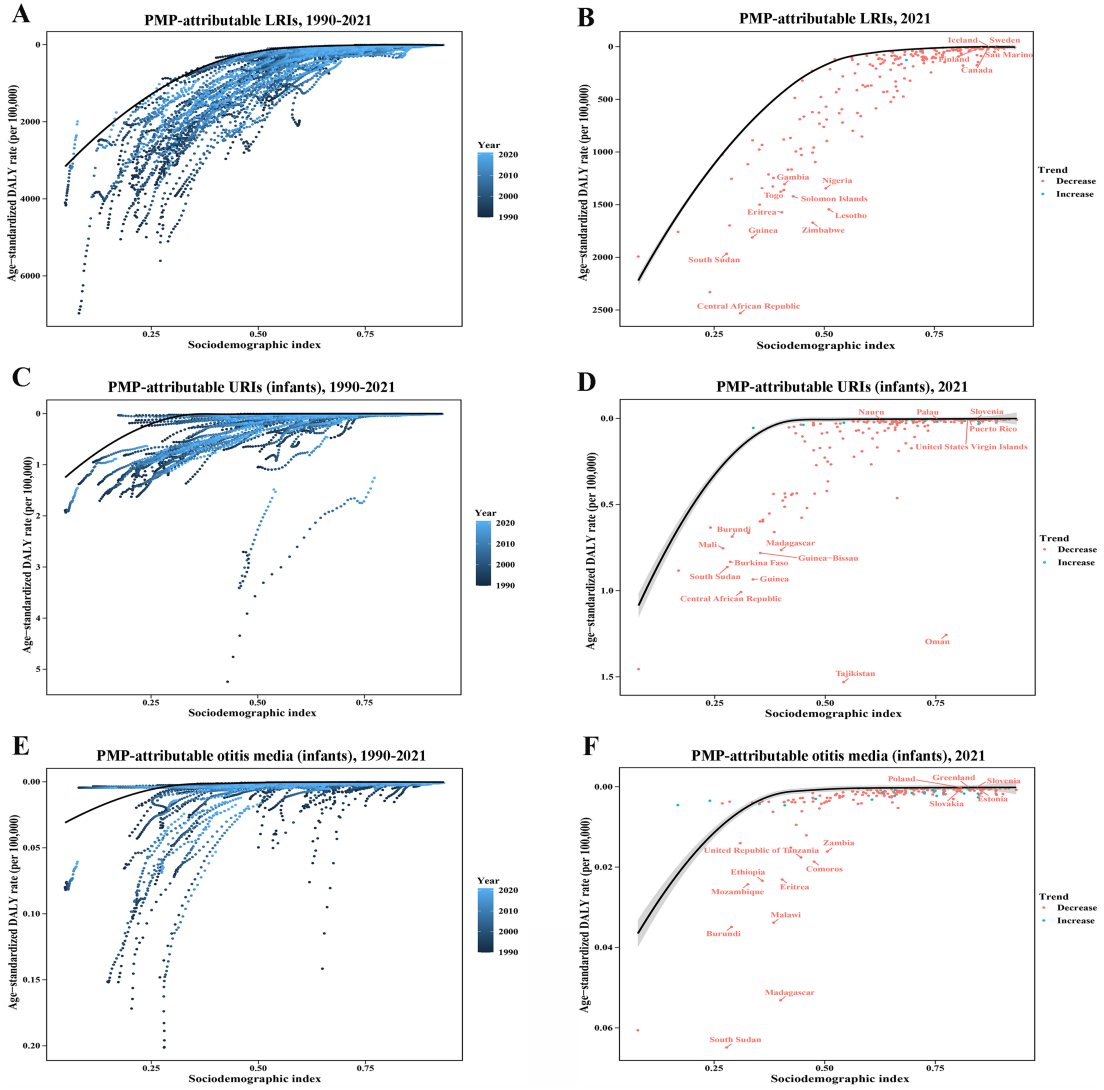
Frontier analysis investigating the associations between SDI and PMP-attributable ASDRs of LRIs **(A,B)**, infant URIs **(C,D)**, and infant otitis media **(E,F)** across 204 countries or territories. In panels **(A,C,E)**, the transition from dark blue (1990) to light blue (2021) illustrates the change over time. In panels **(B,D,F)** each point represents a specific country or territory in 2021. The black line denotes the anticipated ASDR based on SDIs across all countries and territories, and the top 15 countries and territories with the largest deviations from the frontier are highlighted in red. Shaded areas denote 95% CIs.

A frontier analysis was performed to evaluate the potential for improvement in the PMP-attributable ASDRs for LRIs, infant URIs, and infant otitis media, accounting for national and regional SDI levels. The PMP-attributable ASDRs for LRIs, infant URIs, and infant otitis media tended to decrease with increasing SDI (Figure 3). The countries or territories with the largest effective distance in ASDR for PMP-attributable LRIs, characterized by disproportionately high ASDRs relative to their sociodemographic resources, included the Central African Republic, Zimbabwe, Lesotho, and Eritrea (Figures 3A,B). For PMP-attributable URIs in infants, countries or territories such as Tajikistan, Oman, Central African Republic, and Guinea demonstrated the largest effective distances (Figures 3C,D). Similarly, for PMP-attributable otitis media in infants, the countries or territories with the largest effective distances included South Sudan, Madagascar, Malawi, and Burundi (Figures 3E,F).

### Cross-country inequality analysis

**Figure 4:**
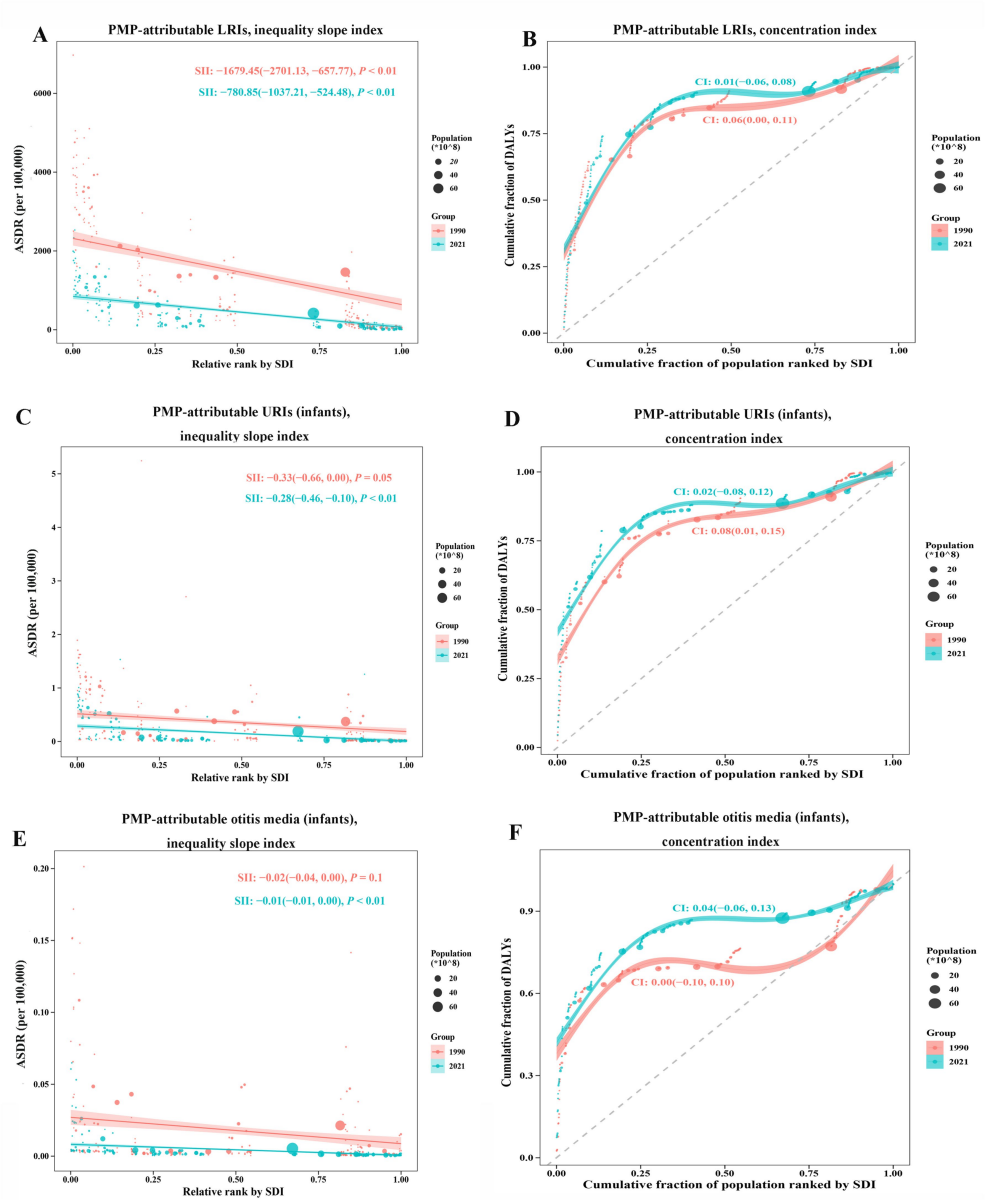
SDI-related health inequality regression curves and concentration curves for PMP-attributable ASDRs of LRIs **(A,B)**, infant URIs **(C,D)**, and infant otitis media **(E,F)** worldwide, from 1990 to 2021. Panels **(A,C,E)** display the slope index of inequality (SII), showing the correlation between the SDI and ASDR for each condition. Each point represents one country or territory, with the size reflecting the population. Panels **(B,D,F)** display the concentration index (CI), which measures relative inequalities by comparing the area under the Lorenz curve to the equality line, reflecting the alignment between the distribution of DALYs and the population stratified by the SDI. Data from 1990 is shown in red, while data from 2021 is represented in green.

Significant absolute and relative inequalities in the burden of LRIs attributable to PMP were observed across countries or territories with varying SDIs, with lower SDI-associated countries and territories disproportionately shouldering a greater burden. The slope index of inequality (SII) revealed that absolute inequalities in ASDRs between the highest and lowest SDI-associated countries or territories. The SII decreased from −1679.45 (95% CI: −2701.13 to −657.77) in 1990 to −780.85 (95% CI: −1037.21 to −524.48) in 2021 (Figure 4A). Similarly, the concentration index was 0.06 (95% CI: 0.00 to 0.11) in 1990 and decreased to 0.01 (95% CI: −0.06 to 0.08) by 2021. These findings revealed that a decrease in both absolute and relative inequalities from 1990 to 2021 (Figure 4B).

For PMP-attributable URIs in infants, the SII decreased from −0.33 (95% CI: −0.66 to 0.00) in 1990 to −0.28 (95% CI: −0.46 to −0.1, *p* < 0.05) in 2021 (Figure 4C). Similarly, the concentration index declined from 0.08 (95% CI: 0.01 to 0.15) in 1990 to 0.02 (95% CI: −0.08 to 0.12) in 2021, reflecting a reduction in both absolute and relative inequalities (Figure 4D). For PMP-attributable otitis media in infants, the SII declined from −0.02 (95% CI: −0.04 to 0.00) in 1990 to −0.01 (95% CI: −0.01 to −0.00, *p* < 0.05) in 2021, indicating a decline in absolute inequalities (Figure 4E). However, the concentration index increased from 0.00 (95% CI: −0.01 to 0.10) in 1990 to 0.04 (95% CI: −0.06 to 0.13) in 2021 (Figure 4F), suggesting an increase in relative inequalities over time.

### Decomposition analysis of DALYs

**Figure 5:**
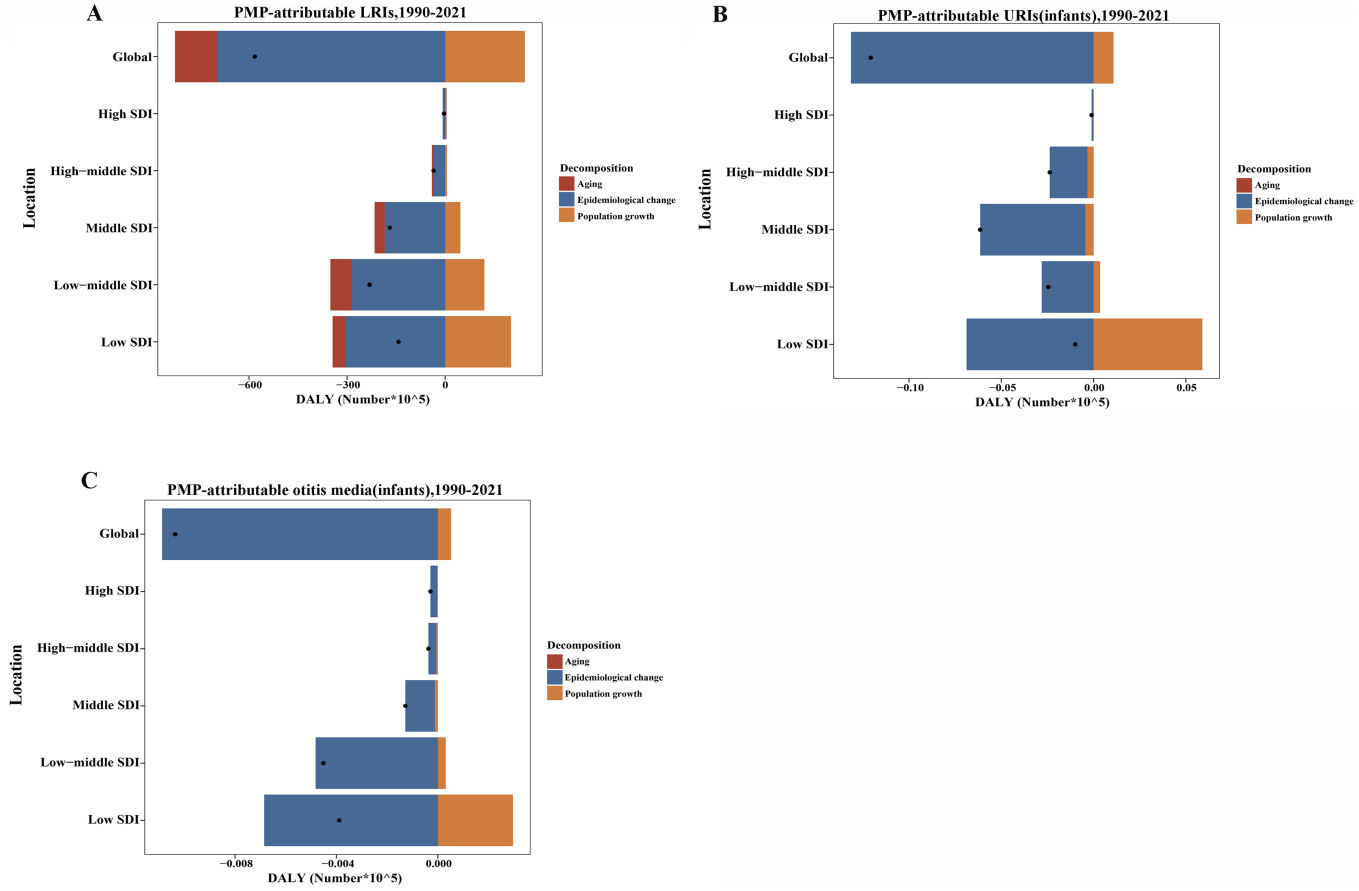
PMP-attributable DALYs for LRIs **(A)**, infant URIs **(B)**, and infant otitis media **(C)**, from 1990 to 2021, decomposed by aging, population growth, and epidemiological changes across SDI quintiles. The black dot represents the overall change resulting from the combined effects of aging, population growth, and epidemiological changes. For each component, positive values indicate an attributable increase and negative values a decrease in DALYs; the absolute value reflects the contribution’s size.

Our study employed decomposition analysis to evaluate the PMP-attributable DALYs of LRIs, infant URIs, and infant otitis media, from 1990 to 2021. This study focused on quantifying the contributions of factors such as aging, population growth, and epidemiological changes to the trends observed in DALYs. Globally, the DALYs of LRIs attributable to PMP demonstrated a declining trend across all SDI regions, with the most pronounced reduction observed in low-middle-SDI regions. Aging and epidemiological changes accounted for 22.53 and 119.37%, respectively, of the global decline in the burden of LRIs attributable to PMP. The impact of population growth exhibited considerable variation across the SDI region, contributing to burden increases of 140.96% in low-SDI regions, 51.89% in low-middle-SDI regions, 27.37% in middle-SDI regions, 15.45% in high-middle SDI regions, and 46.32% in high-SDI regions. The contribution of aging was more substantial in high SDI regions, with specific contributions of 75.48% in high-SDI regions, compared with −28.23% in low-SDI regions, −28.46% in low-middle SDI regions, −18.43% in middle-SDI regions, and −10.84% in high-middle SDI regions. Epidemiological changes significantly reduce the disease burden of PMP-related LRIs globally, particularly in high-SDI regions (−221.8%) (Figure 5A; Table 1).

The PMP-attributable DALYs for URIs in infants also showed a global decline across all SDI regions, with the largest reduction observed in middle-SDI regions. Population growth contributed to a 8.89% increase in the burden, whereas epidemiological changes led to a 108.89% decrease. The impact of population growth was especially prominent in low-SDI regions, contributing 594.78% to the increase in the burden. Epidemiological changes were the predominant factor in reducing the global burden of infant URIs due to PMP, with a particularly high contribution in low-SDI regions (694.78%) (Figure 5B; Table 1).

The PMP-attributable burden of otitis media in infants also largely driven by epidemiological changes, which contributed to 104.98% of the global reduction. However, population growth increased the disease burden, particularly in low-SDI regions (76.11%) and low-middle-SDI regions (6.8%). Notably, epidemiological changes played a decreasing role in the burden globally, especially in low-SDI regions (−176.11%) (Figure 5C; Table 1).

Overall, epidemiological changes emerged as significant contributors to the global reduction in the PMP-attributable burden of LRIs, infant URIs, and infant otitis media, while population growth generally increased the burden, particularly in low-SDI regions. Aging exhibited a variable impact on the burden of LRIs attributable to PMP, contributing to an increased burden in high-SDI regions while reducing the burden in lower-SDI regions.

### Predictive analysis of disease burden to 2031

**Figure 6:**
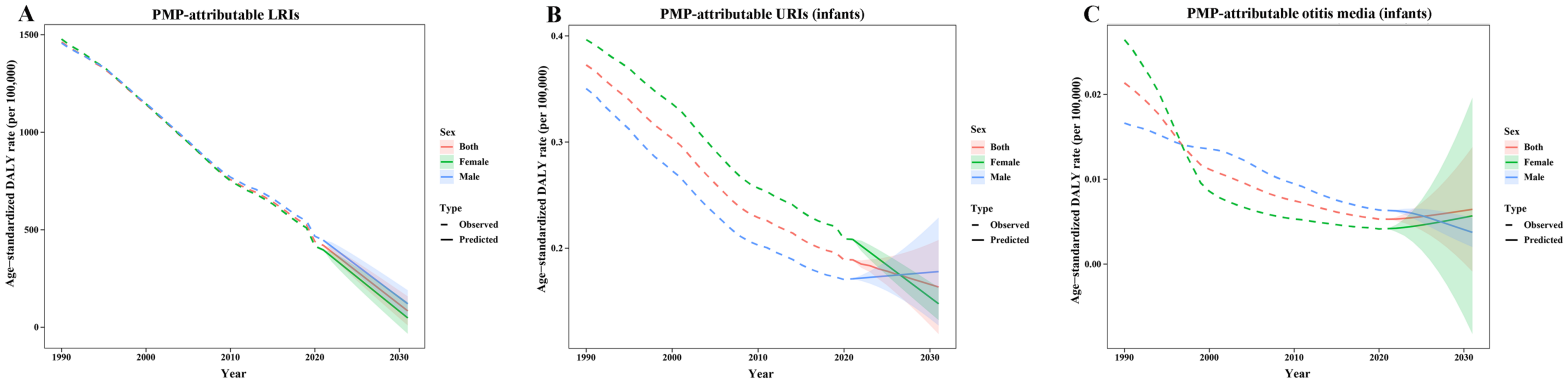
Predicted trends in the PMP-attributable burden of LRIs, infant URIs, and infant otitis media, from 1990 to 2031 based on the ARIMA model. **(A)** The predicted ASDR to 2031 for LRIs attributable to PMP; **(B)** the predicted PMP-attributable ASDR to 2031 for URIs in infants; **(C)** the predicted PMP-attributable ASDR to 2031 for otitis media in infants. Solid lines denote predictions, and shaded bands indicate 95% CIs.

We employed the ARIMA model to predict the PMP-attributable burden of LRIs, infant URIs, and infant otitis media for the period 2022 to 2031. The Ljung–Box test *p*-values, ARIMA model parameters (p, d, q), and corresponding AIC and BIC values are presented in Supplementary Table S4. Using the ARIMA prediction model, the ASDR of LRIs attributable to PMP in the total population is predicted to decrease from 386.47 per 100,000 in 2022 to 83.92 per 100,000 in 2031. Among males, the ASDR is projected to decline from 413.75 per 100,000 to 120.22 per 100,000, and among females, it is projected to decline from 361.72 per 100,000 to 47.92 per 100,000 over the same period (Figure 6A). Using the BAPC prediction model, we projected the global trends in LRIs attributable to PMP from 2022 to 2046. The ASDR is predicted to decline from 345.69 per 100,000 to 63.74 per 100,000, with consistent decreases across all age groups (Supplementary Figures S4, S5). The similarity of projections from both BAPC and ARIMA models supports the robustness of our predictions.

For PMP-attributable URIs in infants, the ASDR of the total population is forecast to decline slightly, from 0.19 per 100,000 in 2022 to 0.16 per 100,000 in 2031 based on the ARIMA model. Among males, the ASDR is predicted to increase marginally, from 0.17 per 100,000 to 0.18 per 100,000, while for females, it is anticipated to decrease slightly from 0.20 per 100,000 to 0.15 per 100,000 (Figure 6B). In the case of PMP-attributable otitis media in infants, the ASDR of the total population is predicted to increase slightly, from 0.005 per 100,000 in 2022 to 0.006 per 100,000 in 2031 based on the ARIMA model. For males, the ASDR is expected to decrease from 0.006 per 100,000 to 0.004 per 100,000, whereas for females, it is projected to increase slightly from 0.004 per 100,000 to 0.006 per 100,000 (Figure 6C).

## Discussion

Our study conducted a thorough evaluation of the global burden of LRIs, infant URIs, and infant otitis media, all attributable to PMP, from 1990 to 2021. Over the past 30 years, the burden of the three disease has significantly decreased. Frontier analysis and cross-country inequalities revealed that many countries or territories with lower SDIs still have significant potential to reduce the burden of these diseases. The decomposition analysis suggested that epidemiological changes were the main contributors to global burden reduction, while population growth was a critical contributor to the increased disease burden in low-SDI regions. Additionally, the ARIMA model indicated a global decline in the PMP-attributable burden of LRIs, and infant URIs, from 2022 to 2031, and a slight upward trend in the PMP-attributable burden of otitis media in infants. These findings highlight substantial regional disparities in the PMP-attributable burden of LRIs, infant URIs, and infant otitis media, and project a rising trend in otitis media, highlighting the need for targeted interventions in high-burden areas.

Prolonged exposure to air pollutants can lead to oxidative stress and pulmonary inflammation (28). Notably, childhood exposure to PM₂.₅ has been associated with adverse long-term consequences, such as developmental impairments, cognitive deficits, and an elevated risk of chronic diseases in adulthood (29). Using updated data from the GBD 2021, we noted a decreasing trend in the PMP-attributable burden of LRIs, infant URIs, and infant otitis media, from 1990 to 2021. This reduction may be attributed to global efforts to improve combustion technology and to implement policies concerning transportation, land use, energy production, healthcare, and social support (30–32). Moreover, significant age-related disparities exist in the burden of PMP-related LRIs. Compared with those of adults, the respiratory systems of children are more vulnerable to air pollution-related respiratory diseases (33). Early infancy is characterized by increased vulnerability, where immune activation can lead to cytokine production, consequently increasing the risk of severe lung inflammation (34, 35). In older adults, extended exposure to airborne particulate matter may exacerbate respiratory conditions (36). The increased susceptibility of older individuals to the respiratory impacts of PMP is largely attributable to age-related declines in immune and pulmonary function (37, 38). Overall, these results align with the findings of our study. Our study suggested that the age group under 5 years and above 95 years had the highest burden of LRIs attributable to PMP. Therefore, managing PMP exposure is crucial for improving health outcomes in children and older adults. This requires enhanced healthcare access, reduced exposure to pollutants, and strengthened public health interventions for these vulnerable groups.

Geographical disparities in the PMP-attributable burden of LRIs, infant URIs, and infant otitis media, are evident across different countries or territories. The most notable reductions in the PMP-attributable burden of LRIs, and infant URIs occurred in East Asia, particularly in China, while the largest decline in PMP-attributable otitis media in infants was observed in Central Europe, notably in Poland. In China, the summary exposure values (SEVs) for household air pollution and ambient particulate matter pollution decreased between 2015 and 2021, probably due to the implementation of a coal ban for residential energy use in areas surrounding Beijing (39). China’s Coal Ban Policy, one of the most extensive air pollution control initiatives worldwide, significantly reduced PM2.5 concentrations, achieving a 4.74 μg/m^3^ decrease in 28 targeted cities (40). Despite progress, low-SDI regions continue to bear the highest PMP-attributable burden of LRIs, infant URIs, and infant otitis media. Particulate matter pollution was identified as the second most significant risk factor in both the low-and middle-SDI regions (11). Our decomposition analysis indicated that population growth plays a more significant role in the disease burden of low-SDI regions. This disproportionate burden of low-SDI regions is largely attributed to their reliance on traditional solid fuels and stoves, rapid population growth, and limited access to clean cooking fuels (41, 42). The World Health Organization (WHO) and the United Nations should place greater emphasis on enhancing healthcare support and medical guidelines in these countries or territories.

As global health continues to evolve, cross-country inequality analysis has become a key approach for identifying health disparities across sociodemographic strata. Our findings revealed a reduction in both absolute and relative inequalities in the PMP-attributable burden of LRIs and infant URIs, from 1990 to 2021, potentially reflecting the improvements in clean energy access, pneumococcal vaccination coverage, and air pollution control policies—particularly in low- and middle-SDI regions (4, 43–45). For PMP-attributable otitis media in infants, absolute inequality declined modestly over time, while the slight increase in concentration index with 95% CIs crossing zero, indicating a non-significant drift in relative inequality toward higher-SDI regions. The SII (absolute inequality) remained negative and decreased, suggesting that absolute burden is still higher in low-SDI regions but the gap narrowed. In most high-income countries, pediatric societies publish acute otitis media (AOM) guidelines that emphasize accurate diagnosis, risk-factor reduction (ie, reducing exposure to cigarette smoke, or promotion of breastfeeding), and routine childhood immunization. By contrast, guidelines are scarce in low- and middle-income countries (Afghanistan; reducing exposure to tobacco smoke) (46). Collectively, these results emphasize the need for equity-driven public health strategies to reduce the burden of PMP-attributable diseases across developing and developed regions.

We projected disease burden trends over the next 10 years using ARIMA model. The PMP-attributable burden of LRIs and infant URIs, are expected to decline through 2031, with a more pronounced reduction observed among females. This trend likely reflects the combined impact of strengthened air pollution control policies, expanded vaccination coverage, and global health initiatives. It has been reported that males are more susceptible to LRIs and females are more frequently affected by URIs, which may be due to anatomical differences in the respiratory tract (47). Although girls initially exhibited a higher PMP-attributable URI burden, projections suggest a more pronounced decline among girls compared to boys, potentially due to a greater relative benefit from gender equity in healthcare interventions. Conversely, a slight increase in the projected burden of PMP-attributable URIs among boys after 2021 may reflect lower antibody production after infection and vaccination compared to girls (48), as well as diminishing benefits from male-focused healthcare policies. For PMP-attributable otitis media in infants, a slight increase in the burden is projected, particularly among girls. Unlike URIs, its management relies heavily on clinical visits and antibiotic use (49). Acute otitis media drives substantial antibiotic prescriptions worldwide (50). Overdiagnosis frequently lead to antimicrobial resistance (AMR) (51), which may increase the disease burden of otitis media through higher recurrence rates, and treatment failures. Evidence suggests that females may experience longer delays in initial antibiotic administration (52), potentially leading to more health-seeking behaviors. Moreover, the rising challenge of antimicrobial resistance (53), and potential overdiagnosis (54), may further contribute to the rising burden, disproportionately affecting girls. In response, the WHO issued the Global Action Plan on AMR (55), and most guidelines recommend a watchful waiting strategy with judicious antibiotic use to reduce AMR (56). Notably, the widening confidence intervals in the projections of PMP-attributable URIs in boys and otitis media in girls indicate considerable uncertainty, highlighting the need for further sex-specific research in pediatric environmental health. Although the ARIMA model offers useful projections based on historical data trends, it does not account for evolving healthcare strategies, economic developments, or risk factor dynamics. Therefore, these estimates should be updated periodically in response to new data and policy changes.

Our study has several limitations due to the reliance on the GBD database, particularly due to restricted data availability in less developed regions (6, 57). Estimates for these regions were derived through mathematical modeling or relied on data from other countries, potentially introducing uncertainty into our findings (58). Second, data on childhood URIs and otitis media attributable to PMP are only available for infants, which may underestimate the burden across all age groups. Finally, our study presents data on the PMP-attributable burden of LRIs, infant URIs, and infant otitis media, encompassing periods before and after the COVID-19 pandemic. To evaluate potential COVID-19 pandemic-related influence, we fit ARIMA models to 1990–2019 data to “forecast” 2020–2022 (COVID-19 years) and compared them with observed values (Supplementary Figure S5; Supplementary Table S5). Relative to forecasts, PM-attributable ASDRs for LRIs (both sexes) were observed 440 and 420 per 100,000 versus 499 and 472 predicted, both observations falling outside the 95% prediction intervals. For PM-attributable URIs in infants, the observed rate was 0.189 per 100,000 (predicted 0.194), outside the 95% prediction intervals in 2020 and within it in 2021. For PM-attributable otitis media in infants, observed rates were remained within prediction intervals. These deviations in PM-attributable LRIs and URIs likely reflect improved air quality and reduced pathogen transmission from non-pharmaceutical interventions and reduced mobility, whereas otitis media changed little, potentially due to antimicrobial resistance, and disrupted vaccination. Despite the COVID-19 shock, our scenario analysis exhibits persistence of downward trend overall, with a slight rebound risk in PM-attributable otitis media in infants. Furthermore, due to the lack of data on PMP concentration changes, our decomposition of DALY trends could not separately quantify the effect of PMP exposure variation, highlighting the need for future studies incorporating external data sources.

In conclusion, the PMP-attributable burden of LRIs, infant URIs, and infant otitis media, declined globally from 1990 to 2021, while projections indicate a rising trend in otitis media. Nevertheless, regional disparities in the burden of these three diseases continue to represent a significant public health challenge. To address this, more effective, equity-oriented, and region-specific strategies are needed to reduce the future burden.

## Data Availability

The datasets presented in this study can be found in online repositories. The names of the repository/repositories and accession number(s) can be found in the article/Supplementary material.

## Footnotes

1
https://www.healthdata.org/gbd/methods-appendices-2021/lower-respiratory-infections-lri-0

2
https://www.healthdata.org/gbd/methods-appendices-2021/upper-respiratory-infections-0

3
https://www.healthdata.org/gbd/methods-appendices-2021/otitis-media-0

4
https://www.healthdata.org/gbd/methods-appendices-2021
