# Correction: Global burden of disease 2021: particulate matter pollution–attributable burden of upper respiratory infections, otitis media, and lower respiratory infections

**DOI:** 10.3389/fpubh.2026.1830410

**Published:** 2026-03-26

**Authors:** Chaochao Wei, Yue Tang, Yang Wang

**Affiliations:** 1 Department of Pulmonary and Critical Care Medicine, Hainan General Hospital, Haikou, China; 2 Department of Pulmonary and Critical Care Medicine, Hainan Affiliated Hospital of Hainan Medical University, Haikou, China; 3 Department of Oncology, Xiangya Hospital Central South University, Changsha, China; 4 NHC Key Laboratory of Tropical Disease Control, Hainan Medical University, Haikou, China; 5 Department of Design, School of Architecture and Art, Central South University, Changsha, Hunan, China; 6 Department of Geriatrics, The Second Xiangya Hospital of Central South University, Changsha, Hunan, China

**Keywords:** burden, DALYs, particulate matter pollution, lower respiratory infections, upper respiratory infections, otitis media

Affiliation “Department of Design, School of Architecture and Art, Central South University, Changsha, Hunan, China” was omitted for author Yue Tang. This affiliation has now been added for author Yue Tang.

Affiliation “Department of Geriatrics, The Second Xiangya Hospital of Central South University, Changsha, Hunan, China” was omitted for author Yang Wang. This affiliation has now been added for author Yang Wang.

Author Yue Tang was erroneously assigned to affiliation “Department of Geriatrics, The Second Xiangya Hospital of Central South University, Changsha, Hunan, China”. This affiliation has now been removed for author Yue Tang.

Author Yang Wang was erroneously assigned to affiliation “Department of Design, School of Architecture and Art, Central South University, Changsha, Hunan, China”. This affiliation has now been removed for author Yang Wang.

The original version of this article has been updated.

