# Supplementary material for: Global burden of disease 2021: particulate matter pollution–attributable burden of upper respiratory infections, otitis media, and lower respiratory infections
Source: Front Public Health. 2026 Mar 12;14:1666976. doi: 10.3389/fpubh.2026.1666976 (PMC13017860; doi:10.3389/fpubh.2026.1666976)
Supplement: Supplementary file 1 [file Data_Sheet_1.docx]

**Supplementary Table S1. Global, and regional DALYs of LRIs attributable to PMP in 1990 and 2021.**

|  | **1990** | | **2021** | | **1990-2021** |
| --- | --- | --- | --- | --- | --- |
|  | **Number of DALYs cases (95% UI)** | **ASDRs/100000 (95% UI)** | **Number of DALYs cases (95% UI)** | **ASDRs/100000 (95% UI)** | **EAPC (95% CI)** |
| **Global** | 87315640 (20780746-139682971) | 1462.2 (340.59-2338.31) | 29098331 (6988265-48127683) | 420.09 (106.35-693.12) | -3.76 (-3.96--3.55) |
| **Sex** |  |  |  |  |  |
| Female | 43317421 (9399480-69269819) | 1477.42 (316.83-2361.7) | 13584531 (3232196-22475876) | 396.58 (100.47-652.59) | -3.91 (-4.14--3.67) |
| Male | 43998219 (11381267-70695047) | 1457.41 (363.88-2344.13) | 15513800 (3757804-25567361) | 446.36 (112.33-735.71) | -3.61 (-3.80--3.43) |
| **Age** |  |  |  |  |  |
| <5 years | 75898555 (18889699-121484084) | 12242.93 (3047.03-19596.17) | 17938384 (5539219-30061319) | 2725.49 (841.61-4567.4) | -4.39 (-4.69--4.1) |
| 5-9 years | 2291581 (352943-3775017) | 392.71 (60.48-646.93) | 870811 (125477-1459054) | 126.75 (18.26-212.36) | -3.36 (-3.6--3.11) |
| 10-14 years | 824470 (131178-1316522) | 153.91 (24.49-245.77) | 490375 (72004-813507) | 73.56 (10.8-122.03) | -2.13 (-2.30--1.97) |
| 15-19 years | 552187  (85933-874020) | 106.31 (16.54-168.27) | 378214 (54867-627110) | 60.61  (8.79-100.5) | -1.84 (-1.95--1.74) |
| 20-24 years | 449277  (71651-696475) | 91.3  (14.56-141.53) | 351591 (51360-576631) | 58.88  (8.6-96.56) | -1.58 (-1.74--1.43) |
| 25-29 years | 413859  (65824-652566) | 93.5  (14.87-147.43) | 360864 (51878-594466) | 61.34  (8.82-101.04) | -1.46 (-1.67--1.25) |
| 30-34 years | 375117  (59270-591807) | 97.33  (15.38-153.55) | 365389 (51806-594681) | 60.45  (8.57-98.38) | -1.5 (-1.69--1.31) |
| 35-39 years | 406697  (64122-637440) | 115.46  (18.2-180.97) | 399381 (56590-657736) | 71.21  (10.09-117.27) | -1.52 (-1.65--1.4) |
| 40-44 years | 407386  (63756-646088) | 142.2  (22.25-225.53) | 455464 (63976-751831) | 91.05  (12.79-150.29) | -1.66 (-1.81--1.51) |
| 45-49 years | 428549  (68614-672966) | 184.56 (29.55-289.83) | 498975 (69046-820406) | 105.38  (14.58-173.26) | -1.93 (-2.05--1.82) |
| 50-54 years | 531195  (86556-844087) | 249.89 (40.72-397.08) | 603752 (84845-1002905) | 135.7  (19.07-225.41) | -2.07 (-2.19--1.94) |
| 55-59 years | 613077  (97966-965970) | 331.04  (52.9-521.58) | 725504 (103683-1195052) | 183.33  (26.2-301.99) | -1.96 (-2.12--1.79) |
| 60-64 years | 783067 (127266-1235554) | 487.56 (79.24-769.29) | 904240 (129804-1499424) | 282.53  (40.56-468.5) | -1.95 (-2.10--1.81) |
| 65-69 years | 786566 (125817-1257860) | 636.33 (101.79-1017.61) | 1016009 (144711-1686302) | 368.33  (52.46-611.33) | -1.68 (-1.86--1.50) |
| 70-74 years | 813301 (129868-1291939) | 960.65 (153.4-1526.01) | 1082983 (154532-1799291) | 526.13  (75.07-874.12) | -1.67 (-1.8--1.54) |
| 75-79 years | 721707 (111663-1157994) | 1172.45 (181.4-1881.22) | 928015 (127719-1539979) | 703.66 (96.84-1167.67) | -1.71 (-1.84--1.59) |
| 80-84 years | 549345  (85283-891309) | 1552.88 (241.08-2519.54) | 801720 (109412-1344084) | 915.38 (124.92-1534.64) | -1.66 (-1.84--1.48) |
| 85-89 years | 307249  (47827-513113) | 2033.27 (316.5-3395.61) | 529085 (70294-902818) | 1157.18 (153.74-1974.59) | -1.73 (-1.92--1.55) |
| 90-94 years | 124071  (18995-208413) | 2895.34 (443.26-4863.57) | 286339 (37601-501551) | 1600.61 (210.19-2803.62) | -1.74 (-1.93--1.54) |
| 95+ years | 38387  (5793-67495) | 3770.48 (568.99-6629.64) | 111237 (14162-198048) | 2040.93 (259.85-3633.7) | -1.72 (-1.96--1.48) |
| **SDI region** |  |  |  |  |  |
| Low SDI | 27857997 (6496927-45594531) | 3500.38 (758.1-5628.63) | 13573287 (3516054-22172136) | 1141.25 (252.52-1851.51) | -3.41 (-3.59--3.23) |
| Low-middle SDI | 33170575 (8426802-52366120) | 2100.66 (504.98-3314.6) | 10061788 (2612667-16823380) | 588.54 (147.28-980.96) | -3.67 (-3.9--3.44) |
| Middle SDI | 20988435 (4735660-33520147) | 1158.32 (255.12-1844.35) | 4046976 (754905-6824679) | 199  (40.46-336.79) | -5.44 (-5.56--5.32) |
| High-middle SDI | 4422947 (970263-7381910) | 487.61 (107.88-812.87) | 904978 (124824-1574821) | 64.5  (10.02-111.28) | -6.76 (-6.99--6.53) |
| High SDI | 826176 (133752-1494283) | 90.29  (15.31-160.78) | 488348 (55174-902268) | 25.79  (3.14-47.22) | -3.68 (-3.86--3.51) |
| **GBD region** |  |  |  |  |  |
| East Asia | 14399103 (3075511-23132323) | 1330.53 (281.05-2135.49) | 1236282 (185792-2079923) | 100.69  (16.75-169.59) | -9.13 (-9.45--8.81) |
| South Asia | 30327576 (7872941-47789837) | 2124.08 (524.26-3337.14) | 9229015 (2565941-15140442) | 613.07 (170.05-1006.35) | -3.52 (-3.78--3.25) |
| Southeast Asia | 7345504 (1674785-11924795) | 1394.7 (309.7-2244.86) | 1717079 (336152-2986290) | 296.14  (59.1-515.93) | -4.64 (-4.8--4.48) |
| Central Asia | 1243199 (295461-2210842) | 1357.09 (319.22-2415.33) | 319247 (75107-558380) | 331.78  (77.42-579.57) | -4.58 (-4.98--4.19) |
| Central Europe | 305234  (50463-541774) | 299.33 (50.84-531.75) | 105087 (14090-180775) | 63.24  (9.05-109.28) | -4.74 (-4.95--4.53) |
| Eastern Europe | 290201  (55779-533614) | 161.84 (32.89-297.16) | 108845 (13842-203176) | 44.91  (6.2-83.76) | -4.57 (-5.36--3.78) |
| Western Europe | 303453  (36065-599657) | 60.03  (7.33-118.35) | 136650 (16104-258611) | 13.6  (1.66-25.51) | -4.66 (-5.01--4.3) |
| Southern Latin America | 98482  (16511-187034) | 210.3  (34.98-399.18) | 82350  (9437-159495) | 99.55  (11.55-191.27) | -1.57 (-1.89--1.24) |
| High-income North America | 172322  (16565-368494) | 52.07  (5.17-111.05) | 47541  (5054-102661) | 8.3  (0.93-18) | -5.67 (-5.96--5.37) |
| Andean Latin America | 794521 (163164-1291532) | 1756.33 (331.67-2846.2) | 168610 (25720-302191) | 282.69 (43.19-506.1) | -5.56 (-5.76--5.35) |
| Central Latin America | 1137907 (223871-1953011) | 591.73 (110.05-1022.54) | 273738 (46776-495115) | 120.77  (21.58-218.8) | -4.59 (-4.78--4.4) |
| Tropical Latin America | 722489 (127612-1322419) | 501.78 (86.06-920.63) | 208043 (26986-400736) | 87.61  (11.47-167.37) | -4.98 (-5.21--4.74) |
| High-income Asia Pacific | 158103  (16842-354439) | 95.33  (11.32-209.79) | 157614 (17844-306197) | 29.83  (3.46-58.74) | -3.17 (-3.47--2.87) |
| Oceania | 184139  (53099-306126) | 2030.07 (551.45-3361.68) | 183769 (58068-311296) | 1056.22 (304.88-1780.64) | -1.48 (-1.76--1.2) |
| Caribbean | 351323 (103447-575961) | 886.93 (246.54-1455.47) | 173395 (47317-288778) | 408.26 (116.94-671.79) | -1.81 (-2.01--1.61) |
| Australasia | 2712  (121-8479) | 13.57  (0.7-42.46) | 3748  (419-7408) | 6.96  (0.86-13.76) | -1.78 (-2.57--0.99) |
| North Africa and Middle East | 4602509 (1227496-7699957) | 993.39 (248.61-1654.34) | 1202625 (262770-1965042) | 224.66  (47.42-366.95) | -4.25 (-4.44--4.06) |
| Central Sub-Saharan Africa | 2536842 (688521-4270416) | 3104.49 (720.88-5161.74) | 1226307 (292686-2104340) | 1176.06 (234.13-2050.86) | -3.18 (-3.39--2.97) |
| Eastern Sub-Saharan Africa | 10081012 (2253624-16793355) | 3504.47 (715.95-5664.99) | 4102895 (973898-6686238) | 1074.65 (214.76-1700.43) | -3.82 (-3.96--3.68) |
| Southern Sub-Saharan Africa | 776082 (155430-1315317) | 1322.18 (246.53-2250.42) | 522511 (86807-888893) | 725.98 (119.12-1234.44) | -1.21 (-1.75--0.65) |
| Western Sub-Saharan Africa | 11482930 (2687141-19085520) | 3605.77 (774.94-5920.94) | 7892981 (2131011-13577771) | 1340.41 (302.39-2272.97) | -2.89 (-3.14--2.65) |

Abbreviations: LRIs, lower respiratory infections; DALYs, disability-adjusted life years; ASDRs, age-standardized DALY rates; EAPC, estimated annual percentage change.

**Supplementary Table S2. Global, and regional DALYs of infant URIs attributable to PMP in 1990 and 2021.**

|  | **1990** | | **2021** | | **1990-2021** |
| --- | --- | --- | --- | --- | --- |
|  | **Number of DALYs cases (95% UI)** | **ASDRs /100000 (95% UI)** | **Number of DALYs cases (95% UI)** | **ASDRs /100000 (95% UI)** | **EAPC (95% CI)** |
| **Global** | 23781.75  (6477.74-48147.78) | 0.37  (0.1-0.75) | 11708.46  (2800.5-28081.79) | 0.19  (0.05-0.45) | -2.32  (-2.39--2.25) |
| **Sex** |  |  |  |  |  |
| Female | 12205.77  (2740.67-27279.4) | 0.40  (0.09-0.89) | 6231.57  (1247.84-16714.53) | 0.21  (0.04-0.56) | -2.36  (-2.45--2.27) |
| Male | 11575.98  (3379.18-22322.8) | 0.35  (0.1-0.68) | 5476.89  (1366.77-12729.77) | 0.17  (0.04-0.4) | -2.48  (-2.58--2.38) |
| **SDI region** |  |  |  |  |  |
| High SDI | 158.17  (113.27-219.51) | 0.03  (0.02-0.04) | 52.22  (30.74-81.67) | 0.01  (0.01-0.02) | -3.03  (-3.19--2.87) |
| High-middle SDI | 2523.02  (1057.96-3631.58) | 0.29  (0.12-0.41) | 148.64  (102.61-239.23) | 0.03  (0.02-0.04) | -8.45  (-9.04--7.86) |
| Middle SDI | 6780.79  (2659.57-9626.64) | 0.34  (0.13-0.48) | 636.09  (444.99-865.5) | 0.04  (0.03-0.06) | -7.22  (-7.49--6.95) |
| Low-middle SDI | 4721.84  (1281.56-10113.6) | 0.26  (0.07-0.55) | 2265.19  (993.04-4134.87) | 0.12  (0.05-0.22) | -2.28  (-2.43--2.13) |
| Low SDI | 9591.61  (908.81-26493.79) | 0.91  (0.09-2.52) | 8600.98  (1029.5-23510.4) | 0.50  (0.06-1.37) | -1.93  (-2.02--1.84) |
| **GBD region** |  |  |  |  |  |
| East Asia | 6345.15  (1215.73-9960.13) | 0.55  (0.11-0.87) | 144.78  (94.87-254.37) | 0.03  (0.02-0.05) | -10.99  (-11.53--10.44) |
| South Asia | 2705.31  (549.24-5993.51) | 0.17  (0.03-0.37) | 1110.77  (424.31-2094.7) | 0.07  (0.03-0.14) | -2.72  (-2.84--2.6) |
| Southeast Asia | 275.46  (170.03-425.29) | 0.05  (0.03-0.07) | 129.22  (79.14-203.57) | 0.02  (0.01-0.04) | -2.29  (-2.37--2.2) |
| Central Asia | 828.56  (497.72-1429.78) | 0.88  (0.53-1.52) | 443.53  (255.8-741.62) | 0.45  (0.26-0.75) | -2.6  (-3.06--2.13) |
| Central Europe | 27.56  (19.44-37.11) | 0.03  (0.02-0.04) | 3.36  (2.02-5.07) | 0.01  (0-0.01) | -5.56  (-5.75--5.36) |
| Eastern Europe | 496.54  (436.17-565.03) | 0.34  (0.3-0.39) | 20.60  (16.42-25.83) | 0.02  (0.02-0.03) | -9.51  (-10.1--8.91) |
| Western Europe | 47.91  (32.35-72.15) | 0.02  (0.01-0.03) | 17.81  (9.27-30.44) | 0.01  (0-0.02) | -2.95  (-3.07--2.83) |
| Southern Latin America | 12.35  (6.42-21.87) | 0.02  (0.01-0.04) | 5.86  (2.31-10.92) | 0.02  (0.01-0.03) | -1.82  (-2.12--1.53) |
| High-income North America | 46.03  (29.67-70.55) | 0.02  (0.01-0.03) | 18.92  (10.65-30.76) | 0.01  (0.01-0.02) | -2.71  (-2.85--2.57) |
| Andean Latin America | 127.63  (60.99-251.89) | 0.23  (0.11-0.45) | 19.58  (9.94-34.49) | 0.03  (0.02-0.06) | -6.77  (-7.06--6.48) |
| Central Latin America | 1328.60  (1076.61-1659.78) | 0.55  (0.45-0.69) | 84.07  (52.41-128.67) | 0.04  (0.03-0.07) | -8.14  (-8.45--7.83) |
| Tropical Latin America | 103.41  (77.98-135.3) | 0.06  (0.05-0.08) | 27.98  (18.29-40.97) | 0.02  (0.01-0.02) | -4.45  (-4.57--4.32) |
| High-income Asia Pacific | 20.74  (11.23-34.13) | 0.02  (0.01-0.04) | 9.69  (4.97-16.45) | 0.02  (0.01-0.03) | -1.28  (-1.66--0.9) |
| Oceania | 5.52  (2.89-9.88) | 0.05  (0.03-0.09) | 10.03  (5.15-17.35) | 0.05  (0.03-0.08) | -0.11  (-0.15--0.07) |
| Caribbean | 27.89  (10.92-62.23) | 0.06  (0.03-0.14) | 20.88  (8.29-42.83) | 0.05  (0.02-0.11) | -0.55  (-0.73--0.37) |
| Australasia | 1.64  (0.22-3.82) | 0.01  (0-0.02) | 1.60  (0.23-4.11) | 0.01  (0-0.02) | -0.26  (-0.6-0.1) |
| North Africa and Middle East | 580.39  (209.3-1262.99) | 0.11  (0.04-0.24) | 306.68  (172.91-604.03) | 0.05  (0.03-0.11) | -1.62  (-1.86--1.38) |
| Central Sub-Saharan Africa | 1382.72  (128.58-4357.54) | 1.13  (0.11-3.57) | 782.22  (113.6-2831.83) | 0.37  (0.05-1.33) | -3.84  (-4.16--3.53) |
| Eastern Sub-Saharan Africa | 5150.86  (445.85-13333.86) | 1.21  (0.1-3.13) | 4139.40  (422.84-11125.59) | 0.63  (0.06-1.7) | -2.03  (-2.09--1.96) |
| Southern Sub-Saharan Africa | 134.57  (70.53-237.02) | 0.17  (0.09-0.31) | 78.73  (47.38-132.14) | 0.10  (0.06-0.17) | -1.5  (-1.61--1.4) |
| Western Sub-Saharan Africa | 4132.92  (459.51-13630.22) | 0.97  (0.11-3.21) | 4332.76  (601.83-13553.73) | 0.51  (0.07-1.6) | -2.06  (-2.17--1.96) |

Abbreviations: URIs: upper respiratory infections; DALYs, disability-adjusted life years; ASDRs, age-standardized DALY rates; EAPC, estimated annual percentage change.

**Supplementary Table S3. Global, and regional DALYs of infant otitis media attributable to PMP in 1990 and 2021.**

|  | **1990** | | **2021** | | **1990-2021** |
| --- | --- | --- | --- | --- | --- |
|  | **Number of DALYs cases (95% UI)** | **ASDRs/100000 (95% UI)** | **Number of DALYs cases (95% UI)** | **ASDRs /100000 (95% UI)** | **EAPC (95% CI)** |
| **Global** | 1363.14  (512.18-2946.84) | 0.02  (0.01-0.05) | 327.40  (156.27-626.36) | 0.01  (0-0.01) | -4.54  (-4.8--4.28) |
| **Sex** |  |  |  |  |  |
| Female | 814.78  (173.03-2410.96) | 0.03  (0.01-0.08) | 124.99  (56.51-232.05) | 0.00  (0-0.01) | -5.97  (-6.72--5.21) |
| Male | 548.36  (258.39-1234.07) | 0.02  (0.01-0.04) | 202.41  (87.58-484.71) | 0.01  (0-0.02) | -3.29  (-3.47--3.1) |
| **SDI region** |  |  |  |  |  |
| High SDI | 33.25  (21.6-44.03) | 0.01  (0-0.01) | 4.05  (2.12-7.03) | 0.00  (0-0) | -5  (-6.13--3.85) |
| High-middle SDI | 43.54  (28.16-62.61) | 0.00  (0-0.01) | 6.23  (3.13-11.33) | 0.00  (0-0) | -4.39  (-5.08--3.7) |
| Middle SDI | 160.24  (99.15-235.52) | 0.01  (0-0.01) | 32.02  (16.91-58.05) | 0.00  (0-0) | -3.66  (-4.38--2.94) |
| Low-middle SDI | 523.21  (93.48-1586.7) | 0.03  (0.01-0.09) | 71.83  (34.69-133.93) | 0.00  (0-0.01) | -6.12  (-7.12--5.12) |
| Low SDI | 601.83  (223.23-1402.17) | 0.06  (0.02-0.13) | 213.01  (89.49-472.66) | 0.01  (0.01-0.03) | -5.05  (-5.13--4.97) |
| **GBD region** |  |  |  |  |  |
| East Asia | 40.27  (19.74-69.22) | 0.00  (0-0.01) | 7.64  (3.28-14.94) | 0.00  (0-0) | -2.73  (-2.95--2.52) |
| South Asia | 611.29  (75-1995.56) | 0.04  (0-0.12) | 62.49  (29-118.71) | 0.00  (0-0.01) | -7.01  (-8.38--5.62) |
| Southeast Asia | 16.27  (7.82-31.82) | 0.00  (0-0.01) | 9.98  (4.5-18.96) | 0.00  (0-0) | -1.41  (-1.53--1.3) |
| Central Asia | 1.46  (0.83-2.53) | 0.00  (0-0) | 0.82  (0.39-1.47) | 0.00  (0-0) | -2.68  (-3.13--2.24) |
| Central Europe | 38.95  (24.07-57.81) | 0.05  (0.03-0.07) | 0.49  (0.22-0.96) | 0.00  (0-0) | -11.27  (-13.62--8.85) |
| Eastern Europe | 1.87  (0.97-3.34) | 0.00  (0-0) | 0.29  (0.13-0.54) | 0.00  (0-0) | -4.73  (-5.27--4.19) |
| Western Europe | 16.33  (10.36-22.73) | 0.01  (0-0.01) | 2.19  (1.21-3.77) | 0.00  (0-0) | -5.5  (-6.47--4.52) |
| Southern Latin America | 0.87  (0.44-1.53) | 0.00  (0-0) | 0.29  (0.1-0.63) | 0.00  (0-0) | -3.82  (-4.62--3.02) |
| High-income North America | 2.95  (1.81-4.63) | 0.00  (0-0) | 0.85  (0.45-1.49) | 0.00  (0-0) | -3.56  (-3.87--3.24) |
| Andean Latin America | 3.65  (1.25-9.31) | 0.01  (0-0.02) | 1.13  (0.51-2.26) | 0.00  (0-0) | -4.44  (-4.62--4.26) |
| Central Latin America | 30.09  (19.87-41.82) | 0.01  (0.01-0.02) | 2.60  (1.32-4.7) | 0.00  (0-0) | -6.51  (-7.64--5.36) |
| Tropical Latin America | 77.21  (48.49-109.62) | 0.05  (0.03-0.07) | 6.31  (3.61-9.81) | 0.00  (0-0.01) | -5.6  (-7.45--3.71) |
| High-income Asia Pacific | 0.97  (0.48-1.82) | 0.00  (0-0) | 0.44  (0.18-0.92) | 0.00  (0-0) | -0.88  (-1.26--0.5) |
| Oceania | 0.34  (0.15-0.66) | 0.00  (0-0.01) | 0.65  (0.27-1.25) | 0.00  (0-0.01) | -0.01  (-0.04-0.02) |
| Caribbean | 1.62  (0.57-4.41) | 0.00  (0-0.01) | 1.29  (0.49-3.52) | 0.00  (0-0.01) | -0.44  (-0.64--0.24) |
| Australasia | 0.10  (0.01-0.32) | 0.00  (0-0) | 0.07  (0.01-0.2) | 0.00  (0-0) | -0.89  (-1.33--0.45) |
| North Africa and Middle East | 10.43  (4.89-20.37) | 0.00(0-0) | 12.96  (5.85-24.46) | 0.00  (0-0) | 0.36  (0.3-0.42) |
| Central Sub-Saharan Africa | 28.00  (4.16-95.85) | 0.02  (0-0.08) | 12.35  (3.73-40.63) | 0.01  (0-0.02) | -4.59  (-4.76--4.42) |
| Eastern Sub-Saharan Africa | 461.61  (205.01-1061.97) | 0.11  (0.05-0.25) | 171.14  (64.81-409.27) | 0.03  (0.01-0.06) | -4.73  (-5--4.46) |
| Southern Sub-Saharan Africa | 2.69  (1.19-5.15) | 0.00  (0-0.01) | 1.93  (0.81-3.72) | 0.00  (0-0) | -0.98  (-1.11--0.85) |
| Western Sub-Saharan Africa | 16.16  (7.69-30.01) | 0.00  (0-0.01) | 31.48  (14.72-59.57) | 0.00  (0-0.01) | -0.13  (-0.16--0.11) |

Abbreviations: DALYs, disability-adjusted life years; ASDRs, age-standardized DALY rates; EAPC, estimated annual percentage change.

**Supplementary Table S4. The (p, d, q), AIC, and Ljung-Box (*P*) values of the ARIMA model for the PMP-attributable burden of LRIs, infant URIs, and infant otitis media.**

| **Cause** | **Sex** | **Model(p, d, q)** | **AIC** | **BIC** | **Ljung-Box(**P) |
| --- | --- | --- | --- | --- | --- |
| **LRIs attributable to PMP** | Both | (0,1,0) | 244.841 | 247.709 | 0.859 |
|  | Female | (0,1,0) | 250.132 | 252.999 | 0.918 |
|  | Male | (0,1,0) | 240.671 | 243.539 | 0.663 |
| **URIs attributable to PMP(infants**） | Both | (1,2,0) | -292.764 | -289.962 | 0.862 |
|  | Female | (0,1,0) | -281.091 | -278.223 | 0.064 |
|  | Male | (0,2,0) | -310.819 | -309.418 | 0.902 |
| **otitis media attributable to PMP(infants**） | Both | (2,2,0) | -456.47 | -452.267 | 0.021 |
|  | Female | (2,2,0) | -419.226 | -415.022 | 0.121 |
|  | Male | (2,1,0) | -496.782 | -491.046 | 0.218 |

**Supplementary Table S5. ARIMA-based projections (2020–2030) of DALYs for LRIs, infant URIs, and infant otitis media, using GBD 1990-2019.**

| **sex** | **year** | PMP-attributable LRIs | PMP-attributable infant URIs | PMP-attributable infant otitis media |
| --- | --- | --- | --- | --- |
|  |  | **ASDRs /100000 (95% UI)** | **ASDRs /100000 (95% UI)** | **ASDRs /100000 (95% UI)** |
| Male | 2020 | 521.638(514.662-528.615) | 0.171(0.169-0.174) | 0.006(0.006-0.006) |
| Male | 2021 | 498.616(484.04-513.192) | 0.169(0.164-0.1745) | 0.006(0.006-0.006) |
| Male | 2022 | 472.695(446.085-499.304) | 0.167(0.158-0.176) | 0.006(0.005-0.006) |
| Male | 2023 | 444.981(406.273-483.689) | 0.166(0.152-0.179) | 0.005(0.005-0.006) |
| Male | 2024 | 416.16(366.112-466.208) | 0.164(0.146-0.182) | 0.005(0.004-0.006) |
| Male | 2025 | 386.655(326.174-447.137) | 0.162(0.139-0.185) | 0.005(0.004-0.006) |
| Male | 2026 | 356.727(286.678-426.776) | 0.16(0.132-0.189) | 0.004(0.003-0.005) |
| Male | 2027 | 326.537(247.69-405.384) | 0.158(0.124-0.193) | 0.004(0.003-0.005) |
| Male | 2028 | 296.186(209.208-383.164) | 0.157(0.116-0.197) | 0.004(0.002-0.005) |
| Male | 2029 | 265.735(171.197-360.274) | 0.155(0.108-0.202) | 0.003(0.002-0.005) |
| Female | 2020 | 482.169(473.499-490.838) | 0.218(0.216-0.22) | 0.004(0.004-0.005) |
| Female | 2021 | 452.908(435.446-470.371) | 0.216(0.211-0.211) | 0.004(0.003-0.005) |
| Female | 2022 | 422.801(396.238-449.364) | 0.214(0.205-0.222) | 0.004(0.002-0.006) |
| Female | 2023 | 392.06(356.494-427.626) | 0.211(0.199-0.224) | 0.004(0.001-0.007) |
| Female | 2024 | 360.844(316.567-405.121) | 0.209(0.192-0.226) | 0.004(0.000-0.009) |
| Female | 2025 | 329.273(276.666-381.88) | 0.207(0.185-0.229) | 0.004(-0.002-0.01) |
| Female | 2026 | 297.436(236.909-357.963) | 0.205(0.177-0.232) | 0.004(-0.004-0.012) |
| Female | 2027 | 265.4(197.363-333.437) | 0.202(0.17-0.235) | 0.004(-0.006-0.014) |
| Female | 2028 | 233.215(158.059-308.372) | 0.2(0.161-0.239) | 0.004(-0.008-0.016) |
| Female | 2029 | 200.919(119.01-282.828) | 0.198(0.153-0.243) | 0.004(-0.01-0.019) |
| Both | 2020 | 499.416(491.17-507.661) | 0.194(0.192-0.196) | 0.005(0.005-0.006) |
| Both | 2021 | 471.866(454.959-488.774) | 0.192(0.187-0.197) | 0.005(0.005-0.006) |
| Both | 2022 | 443.525(417.405-469.644) | 0.19(0.181-0.198) | 0.005(0.004-0.006) |
| Both | 2023 | 414.557(379.102-450.012) | 0.188(0.175-0.2) | 0.005(0.003-0.007) |
| Both | 2024 | 385.094(340.414-429.774) | 0.186(0.169-0.202) | 0.005(0.003-0.007) |
| Both | 2025 | 355.24(301.573-408.907) | 0.184(0.162-0.205 | 0.005(0.002-0.008) |
| Both | 2026 | 325.078(262.73-387.425) | 0.182(0.155-0.208) | 0.005(0.001-0.009) |
| Both | 2027 | 294.671(223.978-365.364) | 0.18(0.148-0.212) | 0.005(0.000-0.009) |
| Both | 2028 | 264.072(185.378-342.766) | 0.178(0.14-0.216) | 0.005(-0.001-0.01) |
| Both | 2029 | 233.32(146.964-319.676) | 0.176(0.131-0.22) | 0.005(-0.002-0.011) |

**
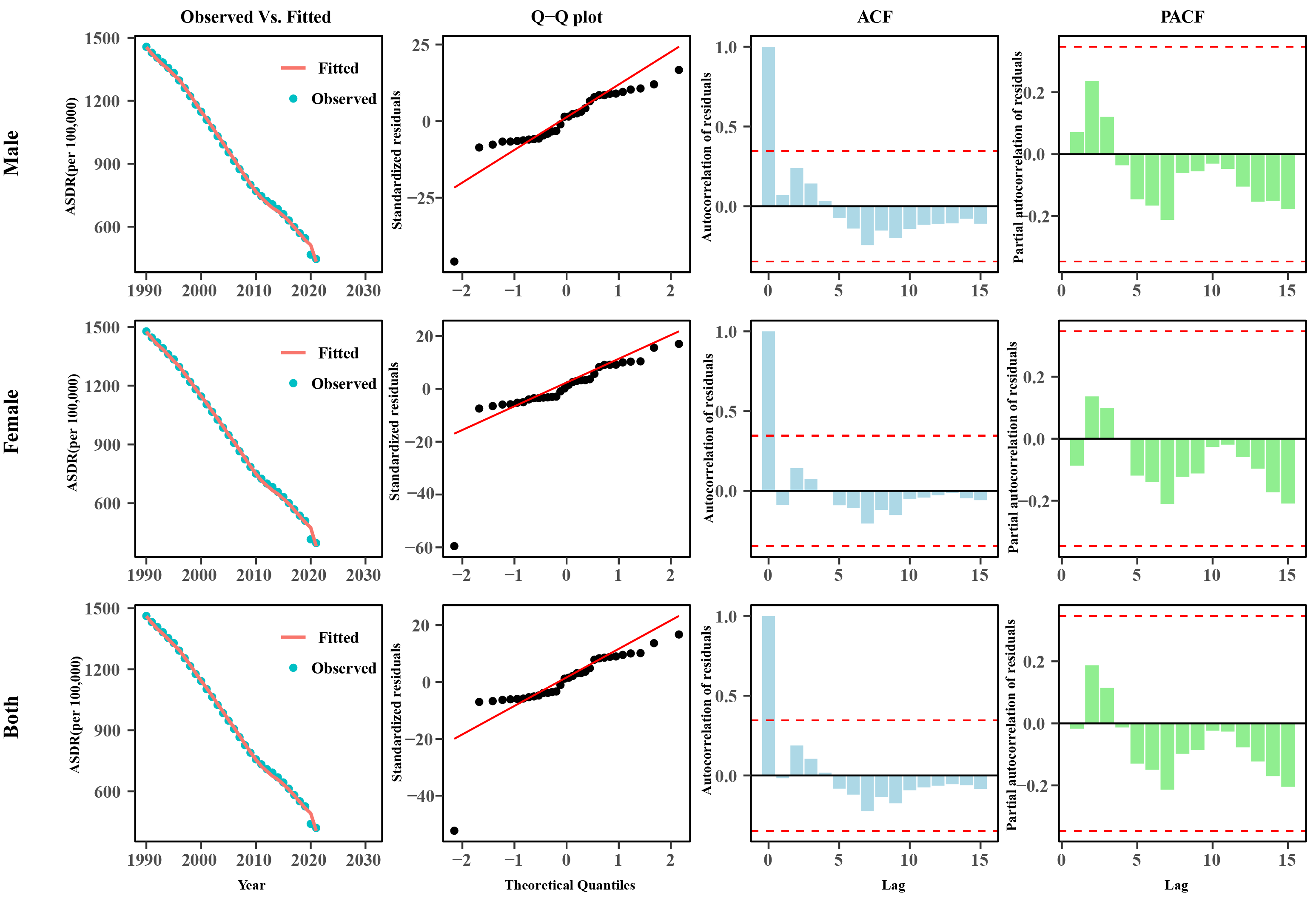
**

**Supplementary Figure S1. ARIMA residual diagnostics for PMP-attributable LRIs.**

**
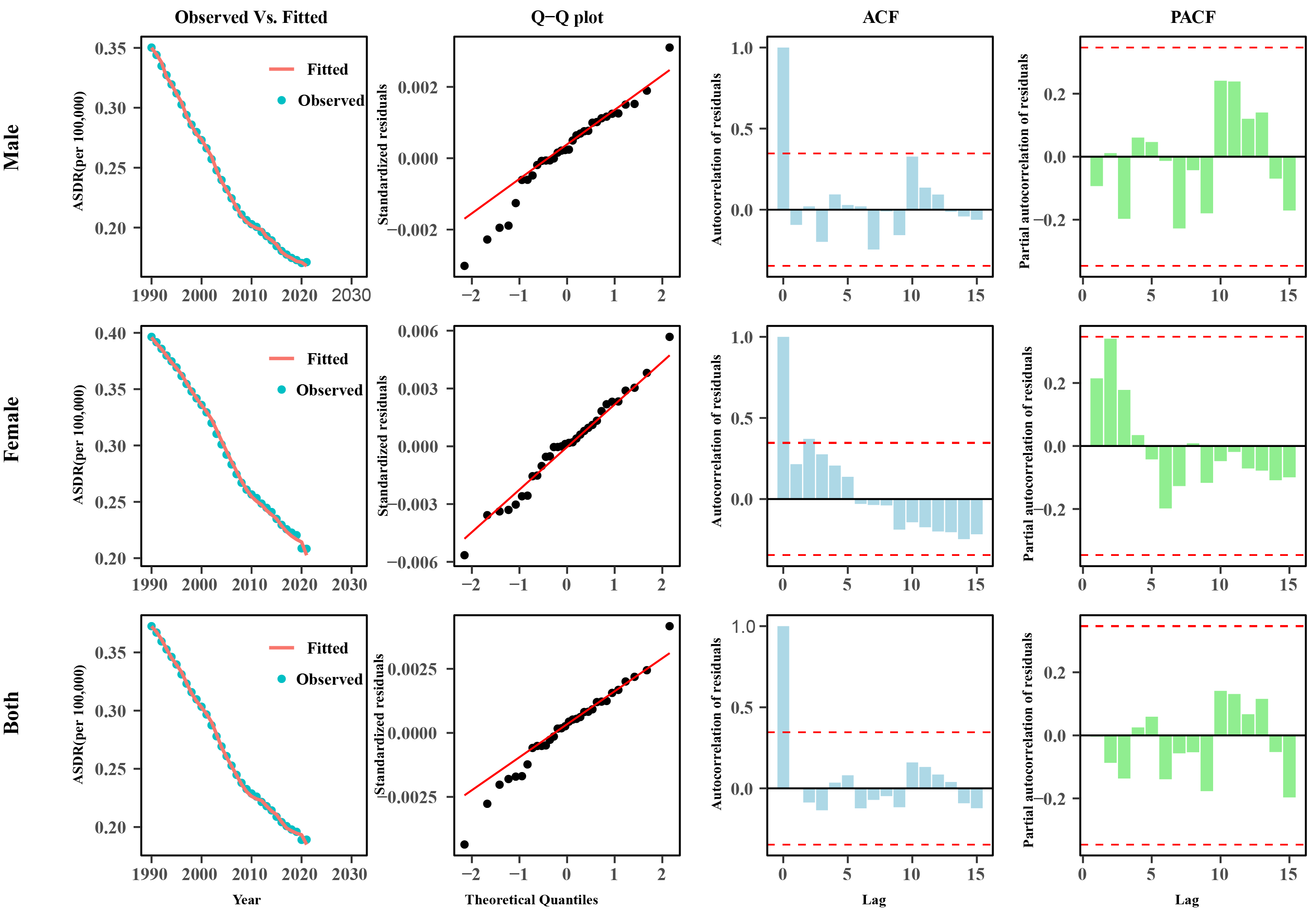
**

**Supplementary Figure S2. ARIMA residual diagnostics for PMP-attributable URIs in infants.**

**
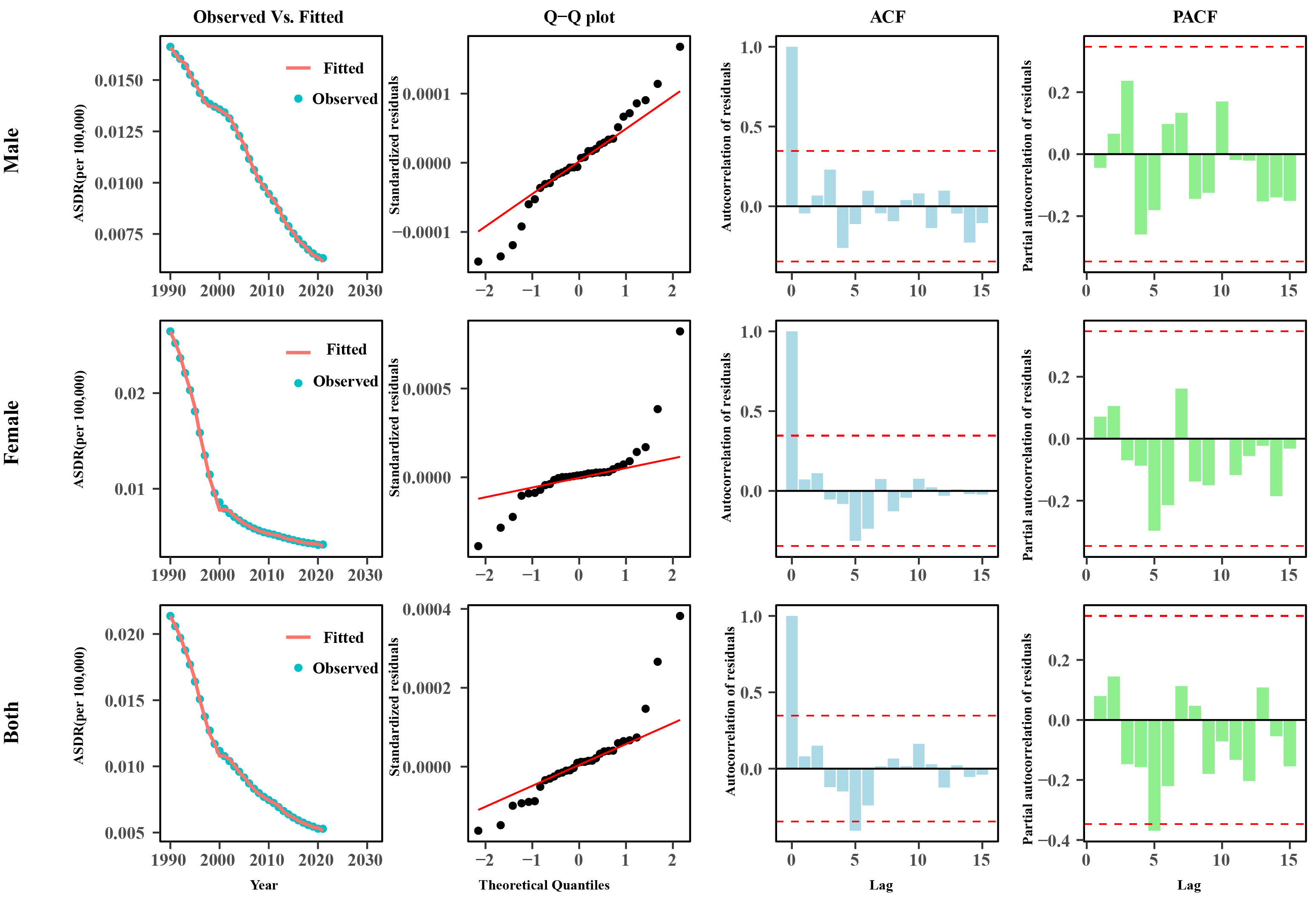
**

**Supplementary Figure S3. ARIMA residual diagnostics for PMP-attributable otitis media in infants.**

**
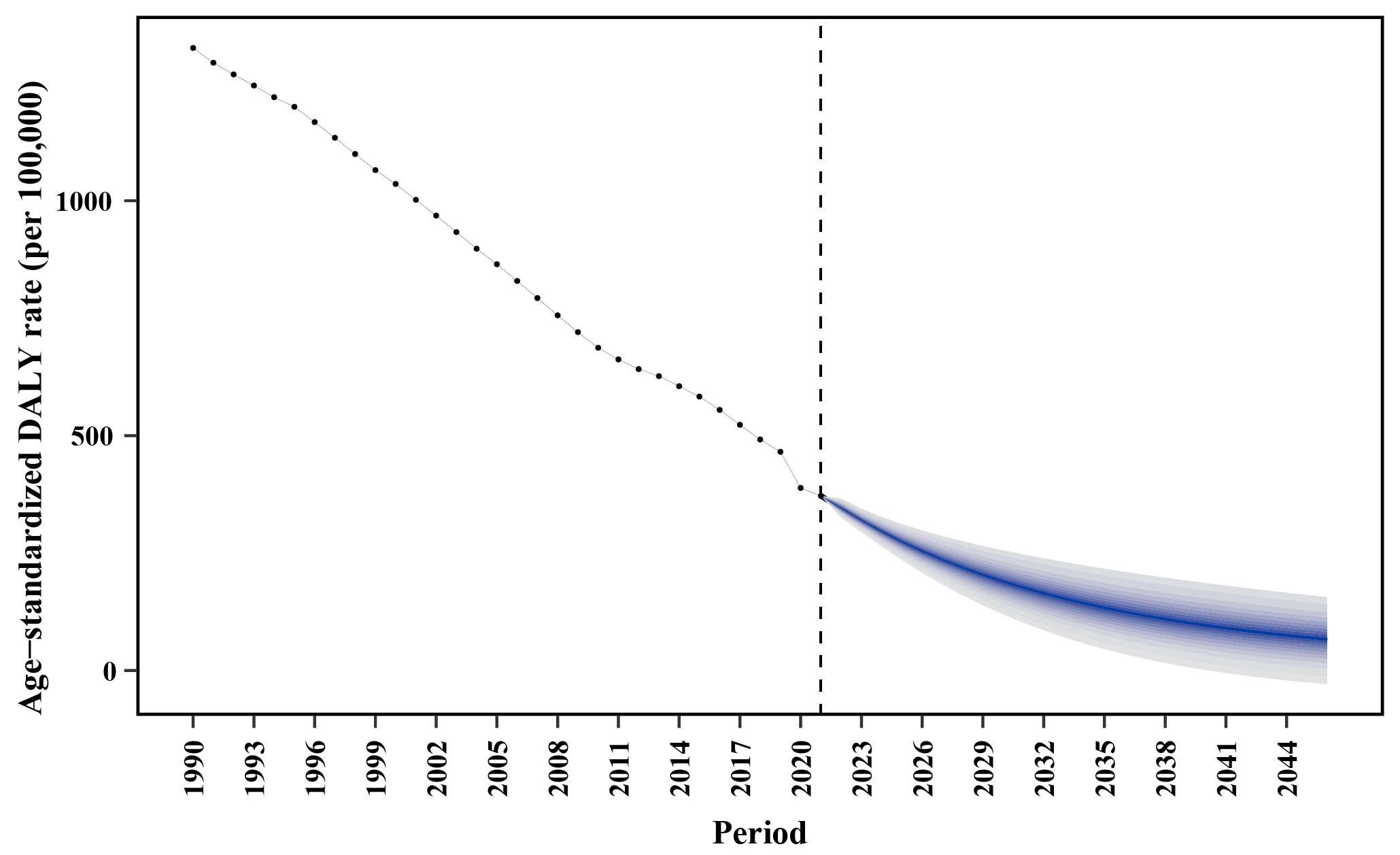
**

**Supplementary Figure S4. Projected burden of LRIs attributable to PMP globally in 2046.**


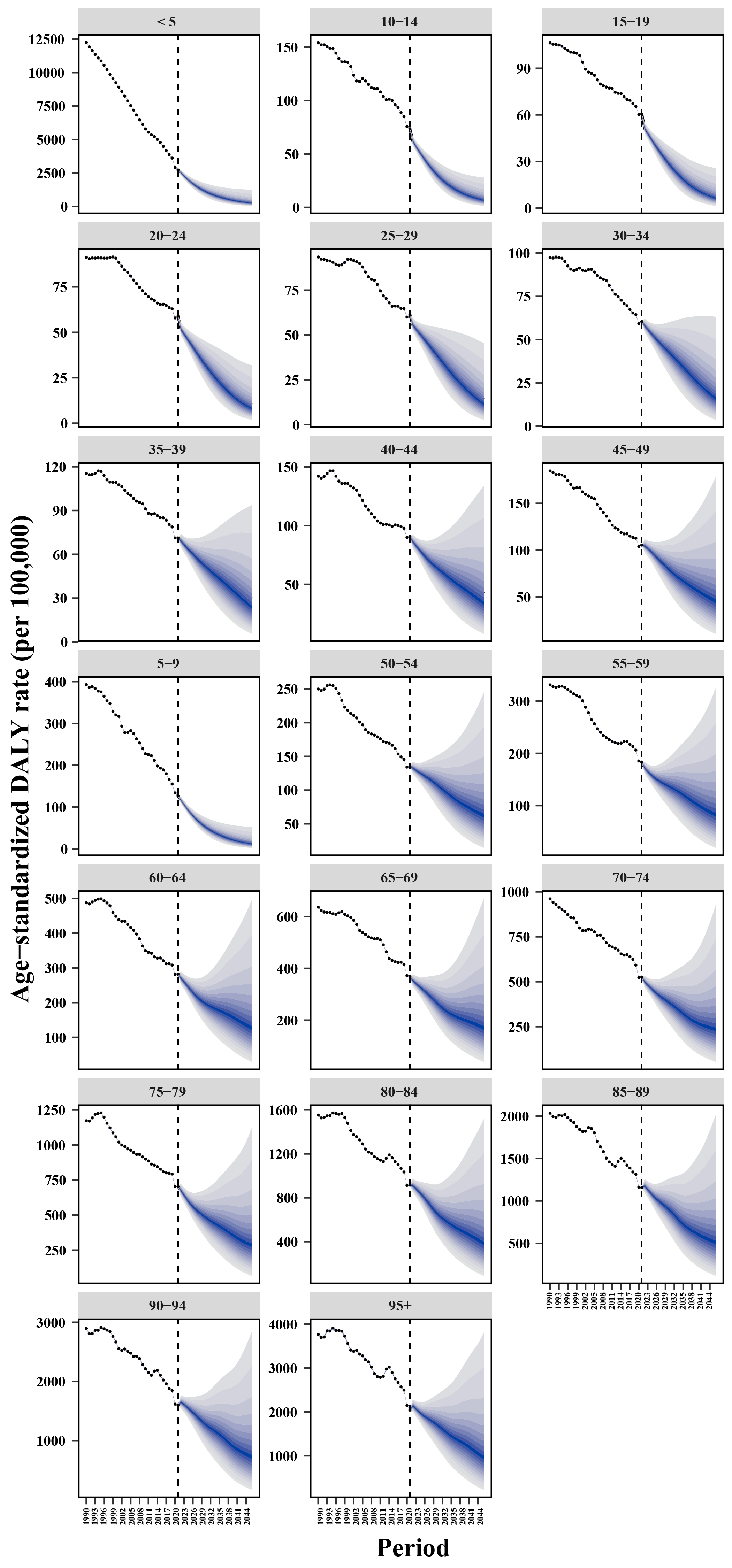


**Supplementary Figure S5. Projected burden of LRIs attributable to PMP globally in different age groups.**

**
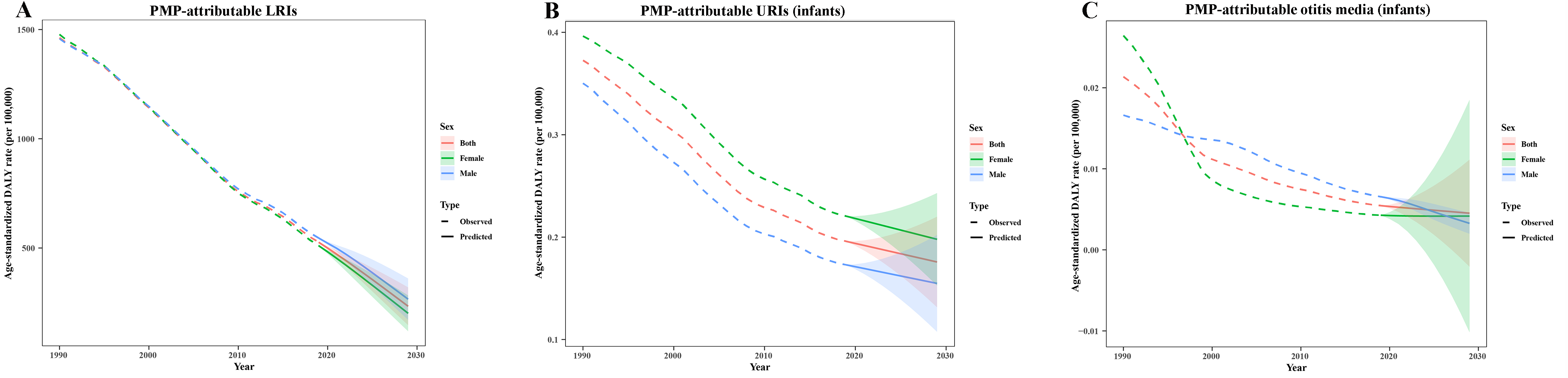
**

**Supplementary Figure S6. ARIMA-based projections of PMP-attributable burden for LRIs, infant URIs, and infant otitis media, using GBD 1990-2019.**
